# Extra-Pair Mating and Evolution of Cooperative Neighbourhoods

**DOI:** 10.1371/journal.pone.0099878

**Published:** 2014-07-02

**Authors:** Sigrunn Eliassen, Christian Jørgensen

**Affiliations:** 1 Department of Biology, University of Bergen, Bergen, Norway; 2 Uni Research, Bergen, Norway; Liverpool John Moores University, United Kingdom

## Abstract

A striking but unexplained pattern in biology is the promiscuous mating behaviour in socially monogamous species. Although females commonly solicit extra-pair copulations, the adaptive reason has remained elusive. We use evolutionary modelling of breeding ecology to show that females benefit because extra-pair paternity incentivizes males to shift focus from a single brood towards the entire neighbourhood, as they are likely to have offspring there. Male-male cooperation towards public goods and dear enemy effects of reduced territorial aggression evolve from selfish interests, and lead to safer and more productive neighbourhoods. The mechanism provides adaptive explanations for the common empirical observations that females engage in extra-pair copulations, that neighbours dominate as extra-pair sires, and that extra-pair mating correlates with predation mortality and breeding density. The models predict cooperative behaviours at breeding sites where males cooperate more towards public goods than females. Where maternity certainty makes females care for offspring at home, paternity uncertainty and a potential for offspring in several broods make males invest in communal benefits and public goods. The models further predict that benefits of extra-pair mating affect whole nests or neighbourhoods, and that cuckolding males are often cuckolded themselves. Derived from ecological mechanisms, these new perspectives point towards the evolution of sociality in birds, with relevance also for mammals and primates including humans.

## Introduction

Females of many socially monogamous species mate with extra-pair males while leaving it to their social mate to provide paternal care [1]. Paternity data exist for more than 200 species of birds, and for 90% of them extra-pair paternity is common [1]. The advantage of such extra-pair mating is obvious for males who may sire additional offspring without the cost of care, but why do females actively solicit extra-pair copulations [2], [3], [4]? After all, a main expectation is that the social male will withdraw his parental care if his share of paternity becomes too low [2] – why would females risk that [5]? Using evolutionary modelling, we show that females who mate with neighbours incentivize males to cooperate towards public goods. From a male perspective, multiple mating and paternity uncertainty imply that their offspring may be spread across several neighbouring nests; this makes it beneficial to focus on the safety and productivity of the entire neighbourhood rather than monopolizing resources for their own social nest. Since many males share that perspective, it is in their self-interest to cooperate with other males to provide such public goods more efficiently. From a female perspective, the benefits of a cooperative neighbourhood may outweigh the risk of lost care from her social mate. Besides, males maintain incentives to stay around, although their paternal investments may be redirected from care provided at their own nest (such as feeding), towards neighbourhood activities (such as vigilance, predator mobbing, or expulsion of intruders).

Our explanation for extra-pair mating in birds is also a new mechanism for the evolution of cooperation, one which may sustain public goods among unrelated males in large groups. One has to be cautious with semantics as the term “cooperation” has different definitions depending on context. In discussions of mating systems, “cooperation” is often used synonymously with “cooperative breeding” [6], a particular mating system in which some sexually mature individuals sacrifice all or some of their reproduction and instead help more dominant individuals to succeed reproductively. In this paper we will use “cooperation” in a broader sense to denote costly and voluntary investments that benefit others (beyond own offspring), and we will in particular focus on cases where collective action is more efficient than multiple individuals acting in isolation.

The discipline of cooperation theory has identified several mechanisms whereby cooperation may evolve, including reciprocity where favours are returned [7], kin selection benefiting relatives [8], mutualism where there is no net cost to cooperation [9], and group-level selection where cooperative groups are more productive and replace selfish groups [10]. Reciprocity has received considerable attention [11] and may be efficient in pairwise interactions [12]. In larger groups, stable cooperation based on reciprocity requires assortative interactions so that cooperative individuals meet more often than by chance [13], [14], agents capable of recognizing cooperative individuals [15], [16], or sanctioning of cheaters [17]. With many players, cooperative benefits need not arise through pairwise interactions but may result from collective investments in a public good; this logic is formalized in public goods games [18]. Here individuals perform costly cooperative acts that produce a public good of greater value than the sum of individual investments, but which anyone in the group may benefit from, regardless of investment. The conflict between the group, which would perform optimally if everyone invested in cooperation, and the individual, who would be better off by exploiting the public good while letting others pay the cooperative investment, is at the heart of the tragedy of the commons [19]. In some cases kin selection may stabilize public goods [20], and in humans sanctioning institutions play a critical role [21]. But in many cases cooperators are neither kin nor do sanctioning institutions exist, from which Clutton-Brock [22] concluded that *“cooperation between unrelated individuals remains a problem”* and May [23] even argued that *“the most important unanswered question in evolutionary biology, and more generally in the social sciences, is how cooperative behaviour evolved and can be maintained in human or other animal groups and societies.”*

Before we detail our mechanism of how extra-pair mating may cause evolution of cooperation it is worthwhile to briefly review the main current explanations for why females in socially monogamous relationships mate multiply. The first class of explanations relate to genetic benefits, often referred to as indirect effects or ‘good genes’. Trivers [24] argued that because females generally invest more in each offspring than males do, they should be choosy about the mate’s quality while males should prioritize the quantity of mates. The ‘good genes’ hypotheses state that since not all females can be paired with the genetically best male, they seek copulations with extra-pair males of superior genetic makeup to increase offspring fitness [3], [25]. A variant focuses on compatibility between the paternal and maternal genome [26], as genetically complementary males may sire heterozygous offspring [27] with potentially higher fitness e.g. through improved immuno-competence [28]. By selecting partners with the right level of genetic complementarity, females may avoid both out- and inbreeding [29]. Theoretical studies suggest that the potential benefits of genetic effects are most likely small [30], that heterozygosity of extra-pair offspring may be overestimated [31], and that beneficial effects to extra-pair half-siblings may be due to maternal effects [32]. Meta-analyses conclude that genetic effects do not provide benefits of the magnitude required to explain its widespread occurrence [33]–[35]. Parker and Birkhead [36] argued that *“given the amount of effort that has been invested (…) and the lack of evidence that females gain indirect benefits, it may be time to consider alternative explanations.”*

In contrast to ‘good genes’ effects, females may mate with extra-pair males to obtain direct or ecological benefits, for example to ensure fertilization of their eggs [37], to obtain nuptial gifts from several mates [38], [39], or to recruit increased paternal care at her nest [2], [40]–[42]. Observations on red-winged blackbirds (*Agelaius phoeniceus*) suggest an even wider cast of female-extra-pair mating. In some populations, females solicit extra-pair copulations after which a territorial male may allow extra-pair females to forage on his territory; he may also defend her nest against predators but not offer similar benefits to other females [43], [44]. Females who included feeding areas outside their social mate’s territory increased the mean and reduced variance in foraging rates [45]. In the group-breeding alpine accentor (*Prunella collaris*) dominant females interrupt copulations of subdominant females and thereby mate with comparatively more males, as a consequence they receive more help and achieve higher offspring survival [46], [47]. Although such conspicuous exchanges of benefits with extra-pair mates are rare [25], we argue that many forms of paternal care potentially are overlooked because they involve investments towards public goods away from the nest. By mating with extra-pair males, females may construct a social network centred at her nest, and which provides benefits to her but in a distributed and diffuse manner. This is in line with Lima’s [48] review of the abundant but poorly explained cooperative behaviours at bird breeding grounds, and may be common in species where offspring are dependent for a prolonged period [49]. Examples of public goods in bird systems include vigilance [50], alarm calls [51], calling networks [52], [53], and predator mobbing [54]. In other taxa, public goods include defence of burrows [55], patrolling of joint territories [56], and sharing of large prey [57].

In this paper we illustrate how the problems of explaining extra-pair mating and evolution of cooperation are two linked questions with a common solution. One direct consequence of extra-pair mating is that it causes paternity uncertainty, which may reduce the risk of infanticide [58], [59]. We extend this logic by noting that paternity uncertainty incentivizes a male not only to abstain from inflicting harm on a potentially direct descendant, but also to cooperate and positively create public goods for the whole neighbourhood since his offspring can belong to any of several broods. From a set of models we derive hypotheses that align with observations of abundant [48] and sex-specific [60] cooperative behaviours at breeding sites, a positive correlation between productivity of offspring mass and rates of extra-pair paternity [61], a strong effect of predation mortality [56], [62], the dominance of neighbours as successful extra-pair sires [63], [64], and aggregation during breeding despite potential for competition and conflict [65].

Our models focus on socially monogamous birds because there exists a rich literature on extra-pair mating for this taxon [1], [33], [34], [62]. Extra-pair mating is also common among fish [49], [66], [67], also live-bearing ones [68]. The literature on mammals more often refers to multi-male mating, which occurs widely for example in rodents [69], [70] and group-living primates [56], [71]. The general theoretical insights thus have relevance for other taxa than birds, so we will return to a general treatment of extra-pair mating in the *Discussion*.

## Models and Results

We use evolutionary models to analyse the influence of extra-pair mating on cooperative behaviours. The models consider two or more socially monogamous breeding pairs, and quantify fitness and selection gradients on traits that determine individual reproductive strategies. We assume no ‘good genes’ benefits but focus only on how the ecological effects of care, provisioning, and protection affect expected offspring survival. We present models for two separate ecological mechanisms, each with specific trade-offs. In our first model, there is competition between males over territories that contain resources required for breeding, and the key trade-off for males is between paternal care at the nest and territorial defence. In a second model, males may engage in collective vigilance and anti-predator behaviours, and the key trade-off is between paternal care and investment in cooperative defence. After showing results from each model we extend both models by assuming a trade-off between an individual’s total reproductive investment and survival, which introduces parental conflict and the possibility of males to abandon nests.

### Territorial competition over resources

#### The ecology of territorial breeding

In many species males defend breeding territories and compete with their neighbours for mates, breeding sites, and food resources [72], [73]. Territorial defence requires vigilance to detect strangers, displays to signal occupancy, and sometimes fighting to expel intruders. The time and resources needed to sustain these activities are often traded off against other activities such as foraging, resting, or parental care [74]. Territorial behaviours may also increase the risk of mortality, as combats may incur injury or fatality, or when vocalizations and displays attract the attention of predators [48], [51].

Territorial behaviours are most conspicuous while borders are fluid and being negotiated [72]. Failing to challenge intruders may lead to territory loss [75] and delayed trade-offs are likely, for example if early investments into establishing a territory has energetic costs that reduce survival or the ability to provide care later in the season [74].

#### The model for resource defence

Our model focuses on bird mating systems where males monopolize resources within their breeding territory, and where these resources can be exploited by the male and female to provision their young. Males can thus invest in territory defence 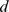
_,_ as well as in offspring care 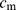, which is directed at the nest and includes provisioning and protection of the young. Females lay a fixed number of eggs at a cost 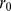 and invest in maternal care 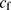 at the nest. For simplicity we assume linear trade-offs between these activities, so reproductive investment is given as 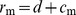 and 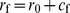 for males and females, respectively. We start with the assumption that 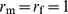, i.e. that males and females have a fixed total investment in reproduction. The amount of resources a male monopolizes is modelled as a tug-of-war, and thus depends on a focal male’s investment in territory defence 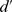 and the defence strategy 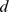 of his neighbour(s), plus competitive pressure 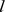 from non-resident floaters. In the simplest case we consider two pairs; then the effect of resource defence on offspring survival is
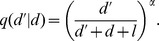



The relative investment in territorial defence hence determines the proportion of resources that the focal male controls and *α* scales the influence of resources on offspring survival. The effect of care on offspring survival depends on contributions from each parent, i.e. 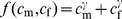. Our argument is not particularly sensitive to the shape of these functions, but we generally assume diminishing returns or a linear effect of investments, i.e. 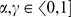. Investments in care benefit offspring directly, whereas the value of resource defence by the male depends also on the defence strategies of neighbouring males. The expected number of surviving offspring *w* is our fitness measure, where 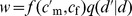 with prime denoting the focal male’s strategy (see Supporting Information S1). Note how care at the nest and access to resources need to be balanced to achieve high offspring survival.

#### Extra-pair paternity and fitness

**Figure 1:**
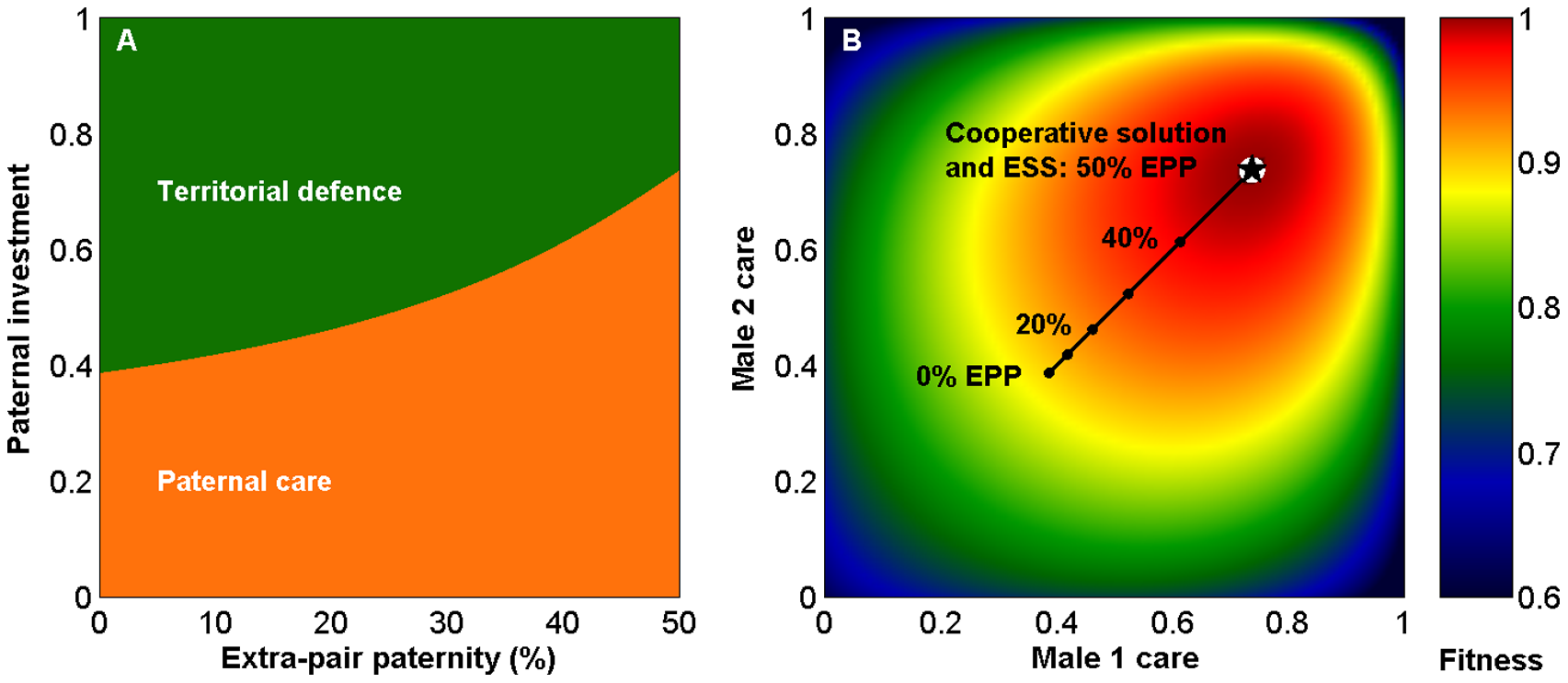
Extra-pair paternity (EPP) favours reduced territorial aggression between neighbours. (A) Evolutionarily optimal male investment strategy in territory defence (green area) versus care (orange) as function of EPP. (B) Average fitness as a function of paternal investment. Increasing EPP levels climb the fitness landscape until the ESS (★) at the cooperative solution (white circle) (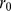 = 0; 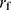 = 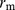 = 1; other parameters as in Table 1).

Consider first a single breeding season and only two neighbouring nests. With no extra-pair paternity, male fitness 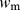 is identical to female fitness 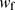. Average offspring survival would be maximized if males cooperated and refrained from aggression, and instead invested heavily in care. In effect, this endpoint of the model corresponds to males defending only external borders of a joint resource territory to keep non-territorial floaters at a distance. This cooperative solution is evolutionarily unstable because a territorial male who aggressively attains a larger share of the resources will have higher fitness. Strong territorial defence will therefore spread and dominate in the population (see Fig. 1).

**Table 1:** Variables and parameters.

Symbol	Description	Value
**Strategy variables**		
*c*_f_	Maternal care (subscript ‘f’ indicates female)	
*c*_m_	Paternal care (subscript ‘m’ indicates male)	
*d*	Male investment in territorial defence	
*k*	Male investment in collective vigilance and defence	
*x*	Rate of extra-pair paternity	
**Functions**		
*f*	Effect on offspring survival of parental care	
*g*	Total benefit for offspring survival of being in a group	
*m*_f_, *m*_m_	Total annual mortality rate	
*q*	Effect on offspring survival of resources defended in territory	
*r*_f_, *r*_m_	Total reproductive investment	
*w*_f_, *w*_m_	Expected fitness, proportional to lifetime production of fledglings	
**Ecological and life-history parameters**		
*a*	Cost of aggregation	0.04
*h*	Rate at which group benefits increase with cooperative investment	0.5
*l*	Competitive pressure from floaters per resource area	0.1
*m*_0_	Annual basal mortality rate	Varied
*m_r_*	Annual mortality rate due to reproductive investment at 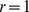	0.1*; 0.25**
*n*	Number of breeding pairs in neighbourhood	Varied
*u*	Summed group investment in cooperation at which the effectof collective defence increases most rapidly	2.0
*α*	Exponent in function 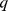 (effect of resources)	0.7
*β*	Exponent in functions 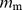 and 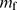 (mortality cost of reproductive investment)	3*; 1**
*γ*	Exponent in function 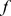 (effect of care)	0.7

*Resource territory model; ** Collective vigilance and anti-predator defence model. (See online Supporting Information S1 for a comprehensive table of variables and parameters.).

**Figure 2:**
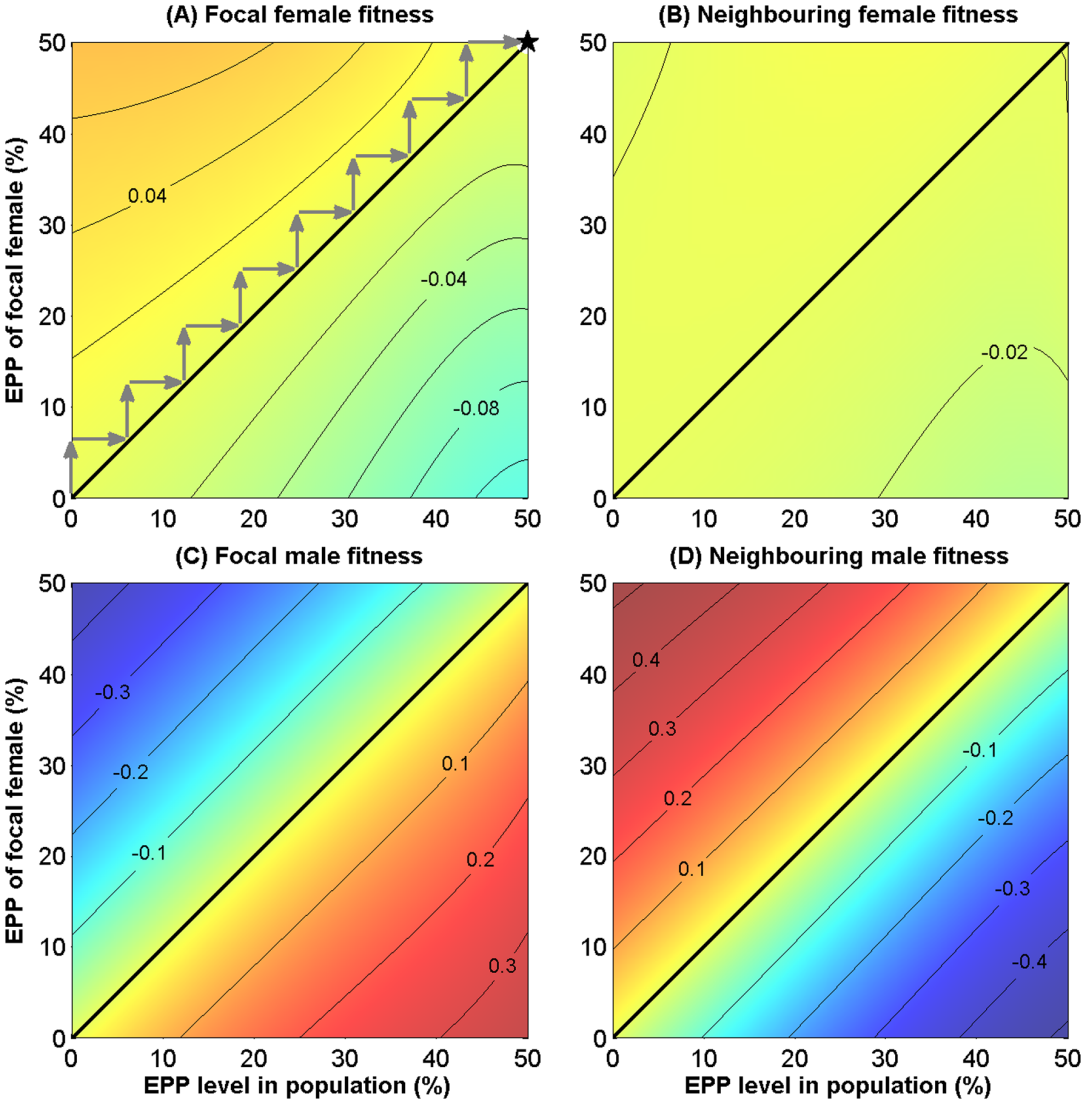
Fitness consequences of female extra-pair mating. (A) Pairwise invasibility plot showing relative fitness of a focal female with a different extra-pair paternity (EPP) level than the population mean. For any EPP level along the *x*-axis, a female with higher EPP than the population mean (above the black diagonal) has higher fitness and can invade and replace the dominant strategy in the population. Arrows show a hypothetical sequence of invasions until the ESS (★) is reached when EPP is 50%. (B) Female extra-pair mating has minor consequences for the neighbouring female, who with these parameters actually benefit too and should therefore not oppose the behaviour. (C) The social mate of the focal female experiences a severe drop in fitness if she increases her level of EPP, and one can expect counterstrategies such as mate guarding to prevent paternity loss in his social nest. (D) As expected, the highest fitness benefit accrues to the neighbouring male, who gets extra offspring that two neighbours will provide the care for. Above sperm, his contribution is to relax territorial defence to allow some resources to flow in the direction of his extra-pair offspring.

Extra-pair paternity may alter this outcome. If each male sires a proportion *x* of the offspring in the neighbour’s nest, male fitness 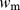 now depends on offspring production both in his social nest 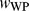 and in the neighbouring nest 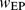:
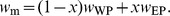



We first assume that males have the same probability of gaining and losing paternity (this assumption is relaxed in the pairwise invasibility plot of Fig. 2); the number of expected offspring is therefore the same but how offspring are distributed across nests has changed. From a male perspective, neighbouring-nest fitness becomes more important when *x* rises. For a male, monopolized resources benefit his offspring in the home nest, but at the same time this takes resources away from his extra-pair offspring in the neighbouring nest. A female who mates with a neighbour therefore incentivizes this extra-pair male to relax territorial defence so that resources flow to his potential extra-pair offspring in her nest. Now facing a less aggressive neighbour, the female’s social mate would gain more resources if he maintained the same territorial behaviour. Because of the trade-off between territorial defence and care, however, his fitness is optimized by reducing aggression, but only so much that he still secures slightly more resources than before. This has the important consequence that it frees time for care. By parallel reasoning, the neighbouring female will benefit from recruiting an extra-pair mate too, which may reduce territory defence even further (see also Supporting Information S2 for an interpretation of the resource territoriality trade-off in terms of a Prisoner’s Dilemma game).

#### Evolutionarily stable strategies and invasion analysis

For a given extra-pair paternity (EPP) rate we find the best male strategy for care and territorial defence using invasion analysis [76], [77] (see also Supporting Information S1). This approach assumes a population where all individuals follow the same strategy (termed “resident”) and considers the growth rate of a rare strategy (referred to as “mutant” and denoted with prime). By making small changes to the strategy for male investment in care and territorial defence we calculate growth rate of this mutant strategy and iteratively replace it with one that does better, until a strategy that cannot be invaded by any mutant strategy is reached; this is considered to be the best male response to a given EPP level. We then repeat this for many EPP values to show how males should optimally respond to different female mating behaviours.

To test whether extra-pair mating can evolve as a female-driven strategy we compare two nests where one of the females follows a mutant extra-pair mating strategy 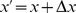 that results in a marginally higher EPP level than in the resident population; if she experiences a net fitness increase it is assumed that genes for that behaviour can spread and establish themselves in the population. When testing mutant EPP strategies, male extra-pair paternity is not symmetrically distributed among the neighbouring males: the social mate experiences increased levels of cuckoldry whereas neighbours benefit from higher EPP. Using the approach described above we find the male strategy that is the best response (denoted with asterisk) of both the within 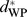 and extra-pair 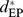 mate of the female mutant. This implicitly assumes that males use female behaviour or other cues to assess within- and extra-pair paternity [78], and that they facultatively adjust paternal care at the nest [2], [79] in response to their mate’s mating behaviour (i.e., males respond by evolved phenotypic plasticity). We then calculate the selection gradient on 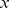, and through repeated iterations make small changes to the resident female strategy in the direction of a positive gradient. Given the model and assumptions it makes, we define the evolutionarily stable strategy (ESS) as the male and female strategy set where mutants of either sex no longer can invade.

#### Results from resource territory model: EPP reduces male investment in territorial defence

Increased levels of EPP select for male strategies with more care and less neighbourhood aggression (a ‘dear enemy’ effect) (Fig. 1A). From a female perspective, reduced territoriality frees time for paternal care, which benefits her offspring. There is thus a positive selection gradient on female extra-pair mating behaviour that leads to higher EPP, provided that males respond to variations in female EPP levels (Fig. 2A). As a result, the mating strategies climb the fitness landscape towards the cooperative solution (Fig. 1B). In the extreme case where the two males have equal proportions of within-pair and extra-pair offspring, each male has the same interest in both broods, resources are equally distributed but defended less aggressively, and the cooperative solution has become the best male response (Fig. 1B).

Despite the benefit of reduced aggression as males gain paternity in several nests, each male has a strong incentive to protect paternity in his own nest (indicated by the strong fitness gradient when moving parallel to the y-axis in Fig. 2C,D). In the model we assume that females fully control the level of EPP, although males that guard their mate may constrain female extra-pair mating behaviour [80], [81]. This may weaken female incentives for engaging in extra-pair mating, reduce EPP levels, and prevent evolution from reaching the cooperative solution.

The effect of reduced territorial aggression is not restricted to a neighbouring pair but may be extended to an entire neighbourhood (see Supporting Information S1). Male incentives for aggressive defence are, however, stronger in larger neighbourhoods because the reduction of one male’s defence will allow several neighbours to grab a larger territorial share; females therefore need to push EPP higher to attain similar benefits.

### Collective vigilance and anti-predator defence

#### The ecology of collective anti-predator behaviours

Breeding birds often engage in cooperative predator defence: they take turns being vigilant, collectively mob predators, or elicit alarm calls that warn others of approaching dangers [48], [54]. A comprehensive study of colonial breeding in bank swallows (*Riparia riparia*) found multiple costs of group living while the only benefit supported by their data was collective anti-predator defence [82]. Cooperation over shared vigilance [83] and collective defence [82], [84] is often more efficient than individual investments and frees time for other activities such as foraging and care. Alarm calls may, however, give away the location of the caller and possibly that of its nest [51], and mobbing may increase risk of injury or death of individuals that participate [54]. Alarm calls and mobbing are thus risky for the individual but beneficial for all group members; an ecological setting similar to public goods games where a common good, shared by a group of individuals, increases with cooperative investments but involves costs to individual cooperators [19].

#### The model for collective vigilance and defence

**Figure 3:**
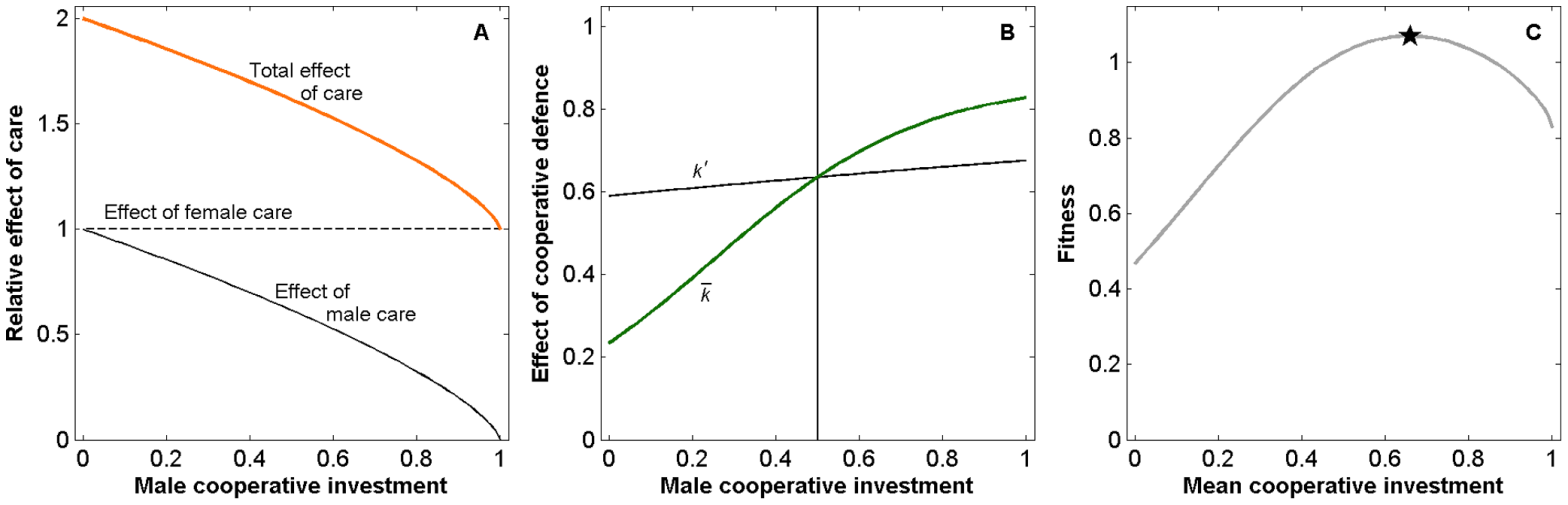
Assumptions of the model for collective predator defence. (A) The relative effect of care on offspring fledging success is the same as for the resource territory model, and is the sum of effects of male and female care. (B) The effect of cooperative predator defence is modelled as a sigmoid function of cumulative investments in the group. The thick green line is the effect if the resident strategy, i.e. the mean level of investment in the group 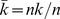, were to change. The thin black line is the effect of one focal individual in a group of 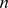 = 8 changing his investment 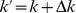 in cooperative defence, assuming that the remaining group members follow the resident strategy with 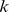 = 0.5. (C) Fitness is the product of the orange and green lines from panels *A* and *B,* respectively, and peaks (★) at intermediate values. (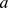 = 0.02; 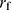 = 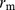 = 1; other parameters in Table 1).

The general assumptions are similar to those of the resource territory model where males and females allocate their reproductive investment between different activities. Here we let male investment in cooperative behaviours *k* conflict with provisioning and care for offspring at the nest 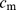, so 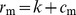. A male who cooperates in collective defence will therefore increase the public good, but at a cost of reduced care in his own nest. We assume that the public good increases with the sum of cooperative investments from all males in a group, and that all group members benefit regardless of their investment. Group members may also experience aggregation costs such as intensified food competition, elevated conflict levels, and higher susceptibility to parasites and diseases. The net group effect 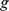 for a group of 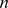 pairs where all males invest 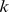 in collective anti-predator defence except for one focal male (denoted by prime) who invests slightly more 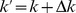 is hence:
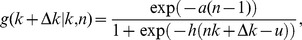
where the aggregation cost increases with the number of neighbours and the interference strength 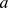. The cooperative benefit increases with the sum of investments in collective defence (Fig. 3), and changes most rapidly when the sum of collective investments is close to 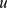.

The evolutionary dilemma is that collective anti-predator defence is more efficient than solitary actions, but each male has incentives to prioritize provisioning and care for offspring in his nest and let others invest in cooperative defence. Again, females can change the evolutionary outcome by extra-pair mating. In a group of *n* pairs, the focal male who invests slightly more in cooperation than the rest, has fitness:
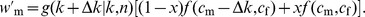



More cooperation (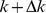) will increase the public good (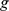) and benefit all his offspring, whereas reduced care (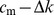) only affects the within-pair offspring in his own nest. As the proportion of extra-pair young increases with higher 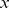, the male experiences the full benefit of a given cooperative investment, but the cost affects only his social nest and is reduced. As before, the fitness of the mutant strategy is related to that of the resident population and the best male strategy is found where no 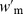 is larger than the fitness 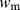 of the resident strategy.

#### Results from collective vigilance and anti-predator defence model: Extra-pair mating increases cooperative investments

**Figure 4:**
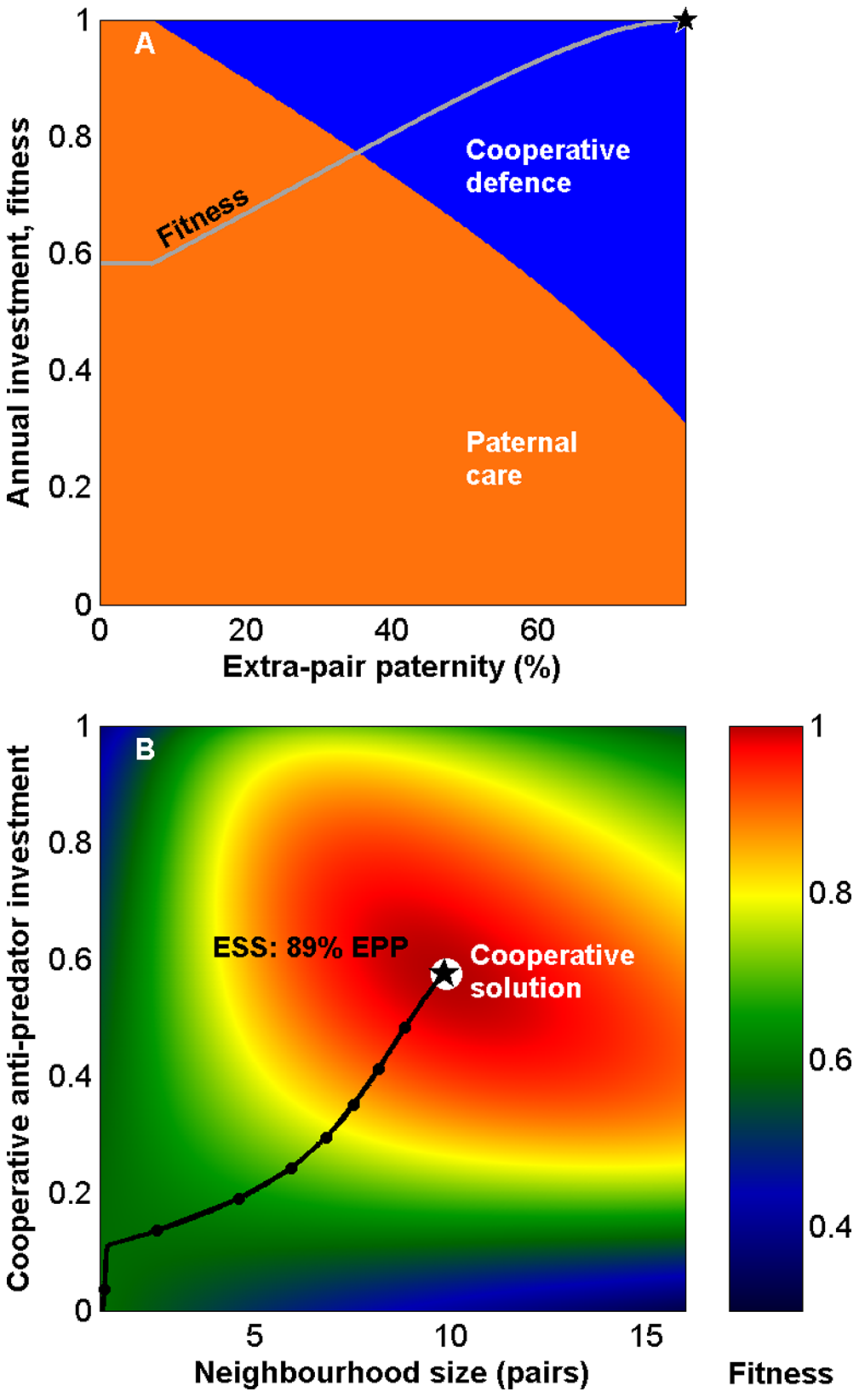
Extra-pair paternity (EPP) promotes cooperative anti-predator defence. Males trade-off paternal care and cooperative predator defence. (A) As EPP increases, males cooperate more because costs of reduced paternal care affect only within-pair young whereas cooperative defence protects all offspring. (group size 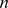 = 5; 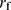 = 1.0 of which egg investment 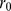 = 0.3; see Table 1 for other parameters). (B) Fitness landscape as a function of neighbourhood size and male cooperative investment. With no EPP the evolutionary outcome is solitary breeding with no cooperation (lower left corner). EPP increases along the black line (dots mark each 10%). Short-lived species reach the cooperative solution (white circle) for high EPP levels.

Extra-pair mating incentivizes males to invest in cooperative behaviours. This happens because the cost in a male’s social nest affects both his genetic offspring and extra-pair young sired by others, whereas his cooperative investment benefits all his offspring independent of location. As the proportion of within-pair offspring in his own nest decreases, the threshold for engaging in cooperation is lowered. The evolutionarily stable male strategy involves higher investments in collective defence and less care directed towards his social nest, which adds up to a benefit to the whole neighbourhood (Fig. 4A). Females benefit from recruiting extra-pair mates because they will cooperatively protect her nest, but this comes at the cost of reduced investment from her social mate as he gains less paternity. Although females receive less help with care, they experience a net fitness benefit because offspring survival increases. As EPP levels increase and trigger higher cooperative investments by males, cooperation can be stable also in groups larger than two pairs and thus become more efficient. Extra-pair mating may thus be a mechanism to extend the social neighbourhood with a positive effect on fitness, as shown by the fitness landscape in the background of Figure 4B where offspring survival peaks at intermediate group sizes and relatively high levels of extra-pair paternity.

### Extending models with trade-offs between current and future reproduction

Males may respond to cuckoldry by decreasing current reproductive investments if alternative reproductive opportunities exist [5], [84]. We include this possibility by allowing males and females to allocate reproductive investments across several breeding seasons. Reproductive investment in one season may influence future reproductive events, for instance by reducing the probability that a parent will survive to the next breeding season [85]–[87]. This trade-off makes the game between the male and the female more pronounced and there is room for parental conflict with potential consequences for future breeding attempts and longevity. We assume that the risk of mortality increases with higher reproductive investments 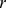, from a baseline mortality 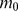:
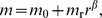
where 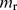 and 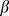 scale the relative cost of current investments. Male and female reproductive strategies affect their expected longevity, and mortality may also reduce current reproductive output if one of the parents dies during the breeding season. If females invest more in reproduction than males, they have lower survival probability, which affects the operational sex ratio and may intensify male-male competition for mates (see Supporting Information S1).

#### Results from extended models: Extra-pair paternity declines with expected longevity

**Figure 5:**
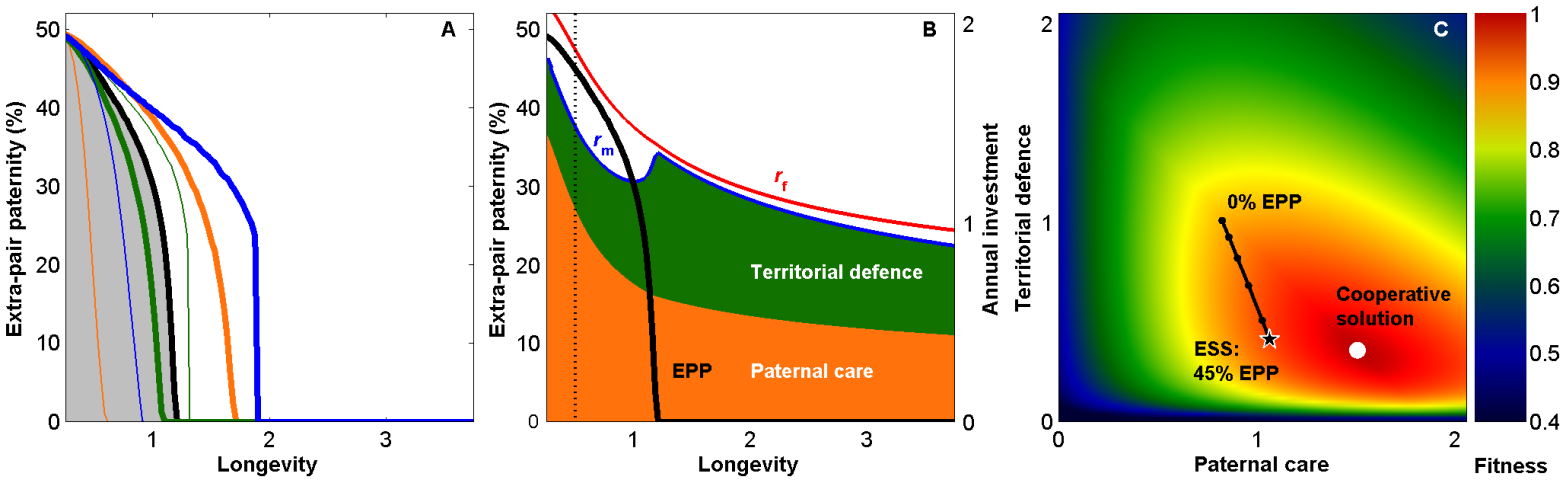
Extra-pair paternity (EPP), territorial defence, and longevity. Annual reproductive investment 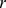 evolves in a trade-off with survival. (A) EPP is common in short-lived species (grey) and drops with increasing life-expectancy (black line, parameters in Table 1). The pattern is robust but the predicted EPP level depends on ecological parameters, e.g., mortality cost of reproduction (green, 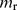 = 0.05 and 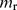 = 0.2 for thin and bold line, respectively; blue, 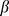 = 2 and 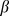 = 4), and the proportion of adult mortality experienced during breeding (orange, 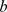 = 0.1 and 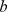 = 0.5). (B) For short-lived species, the ESS involves high EPP, reduced defence, and elevated care. With increasing longevity, the evolutionary outcome is low or no EPP (black line) and more territorial defence. (C) Fitness landscape for combinations of male care and defence strategies in a short-lived species (longevity 0.5 breeding seasons; 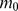 = 1.59). Higher EPP results in less territorial defence, but the evolutionarily stable care strategy (★) is below that of the cooperative solution (○).

**Figure 6:**
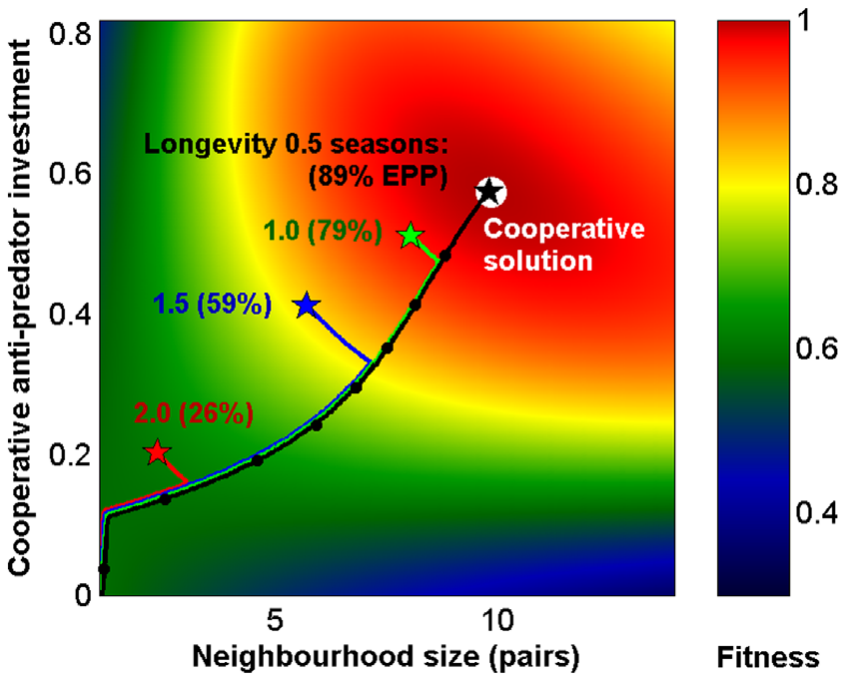
Longevity reduces EPP levels and male-male cooperation. Fitness landscape as a function of neighbourhood size and male investment in cooperative defence. With no EPP the evolutionary outcome is solitary breeding with no cooperation (as in Fig. 4B). As EPP increases along the coloured lines, the best male strategy (★) approaches the cooperative solution (white circle) in short-lived species (dots mark each 10% increase in EPP). In more long-lived species males prioritize future reproduction and high EPP levels are not beneficial to females; the best male strategies are found further away from the cooperative solution (baseline mortality; 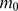 is 1.69 (black), 0.83 (green), 0.49 (blue), and 0.30 (red)).

When longevity increases and future breeding becomes more likely, both males and females evolve reduced annual reproductive investment. Females benefit from EPP only as long as reduced aggression over resources increases male investments in care (resource territory model; Fig. 5, Supporting Information S1) or when the benefits of male-produced anti-predator defence outweigh the costs of reduced paternal care (collective vigilance and defence model; Fig. 6). Males who lose paternities in the home nest reduce their reproductive investment more than others; females therefore need to balance the cost of reduced care against the benefits they can achieve through EPP and male-male cooperation. In model for collective vigilance and anti-predator defence, joint protection from neighbours makes it easy for cheating males to opt out of the cooperative defence by reducing current investments or even abandoning the nest. This is an obvious cost for females and EPP levels are consequently predicted to decline and approach zero as longevity increases (Supporting Information S1), which is in line with observations [62] and general theory [42], [88]. Note that the model does not assume any carry-over effects of reproductive investments, so any surviving individual has the same probability of being mated, which may have implications for the level and stability of cooperation.

## Discussion

In this paper we have presented a new mechanism for the evolution of cooperation towards public goods. The logic is simple. When a male has all his offspring in a single nest, evolution favours reproductive strategies that focus his attention there: by competing with others to maximize his share of resources; or by cheating on public goods and withdrawing from cooperative investments, thereby causing a tragedy of the commons. Female extra-pair mating has the important role of altering the incentives for males: because males potentially have offspring in several nests, natural selection favours males who cooperate towards the productivity and security of the entire neighbourhood. This may include sharing of resources, reducing aggression, being vigilant, alarming of dangers, and defending the neighbourhood rather than the single nest. Cooperation thus evolves from individual male and female self-interests, making the whole neighbourhood safer and more productive.

### Paternity and paternal investment

#### Effects of paternity uncertainty

It has been noted that multi-male mating confounds paternity and thus prevents males from committing infanticide of offspring that are potentially theirs [58]. Hrdy [89] extended this logic in the context of allo-parental care; her focus was how high male mortality in many hunter-gatherer societies makes it unlikely that a paternal care-giver will survive for the whole duration of offspring dependence. Extra-pair mating may then serve a bet-hedging function, as mothers can enlist support from other males in case her partner dies. Stacey [40] suggested a more direct causation, in that females through extra-pair mating may recruit multiple males to help with care for the female’s offspring. This thinking was central also to the eye-opening studies on the intricate and variable mating systems of the dunnock [2], [41]. The common logic is that paternity uncertainty may not only prevent something negative but may also produce something positive. Our approach extends this perspective by showing mechanisms through which neighbourhood cooperation among unrelated males may evolve.

#### Paternal reproductive investment beyond care at the nest

Studies of paternal care in birds have focused on quantifiable male activities at the nest; these typically include brooding, feeding, and offspring protection [2], [90]. Behaviours at the nest need not be the only activities a male engages in to maximize the fitness of his offspring. Lima [48] pointed to many cooperative behaviours at bird breeding grounds that resemble public goods but which have received little attention, including alarm calling [51] and mobbing [54], [82], [91]. These are seldom regarded as parental care investments, but as long as cooperative behaviours benefit potential offspring and are costly to the male in terms of energy, survival, or opportunity, they should be included in the budget of reproductive investment [40], [56].

Although paternal contributions away from the nest are central to our models, it is important to note that the cooperative benefit and its consequences for care differ between our two models. In the model for collective vigilance and anti-predator defence, extra-pair mating causes individual males to provide *less* paternal care at their social nest and instead take part in neighbourhood activities that produce a public good, such as vigilance and mobbing. This is in contrast to the resource territory model, where extra-pair mating causes *more* paternal care at the nest because paternity spread takes away reasons for aggressive resource monopolization.

### Reproductive conflict and the mating game

At breeding grounds, both competition and cooperation with others influence individual pay-offs. The male and female of a mated pair shares a common interest in raising viable offspring, but each may benefit from having the other investing more in care and protection [24]. Among males, the traditional view is that conflict dominates [92], but a consequence of the mechanisms in our models is that cooperation among unrelated males emerges. This brings with it new and sex-specific lines of conflicts, where males may adjust care investments in response to mating access or paternity [2], [41]. Mating with multiple males may be a mating strategy by which females trigger help from additional males [40], and they may hence compete with other females to channel benefits produced by males towards their own nest. Although there is intense competition among males for matings, the game changes once eggs have been fertilized as neighbourhood cooperation may be favoured by individual-level selection.

#### Female mating tactics

Our models show that the level of extra-pair mating may evolve as a female-driven strategy, which is corroborated by several empirical observations: females are observed to actively seek extra-pair copulations in many species [2], [3], [4]; forced copulation is not common in birds [93]; and in one particularly well-studied population of song sparrows (*Melospiza melodia*) the heritability of male extra-pair success is virtually none [94] whereas the proportion of extra-pair young in a female’s brood shows significant heritability [95]. These lines of evidence support the view that extra-pair mating is at least partly under female control, but to what degree may vary even among populations of the same species [96]. Females can also control fertilization through post-copulatory selection and sperm competition [25], including ejection of sperm from previous matings [2]. If females synchronize their fertile period, males experience a stronger conflict between mate guarding and soliciting of extra-pair copulations, which likely makes it easier for females to control their mating activity [41], [97].

#### Male mating tactics

To evolve as a female-driven strategy, extra-pair mating has to channel more benefits towards a female’s nest than achieved by less promiscuous females. This requires phenotypic plasticity in the male response to the shift of paternity distribution, and therefore that they have some information on which to act. In birds, males can rarely recognize their own offspring [78], but they may use information about their mate’s behaviour to assess within-pair paternity. When it comes to extra-pair paternities, it is a safe assumption that males have information about their own extra-pair mating activity and can use that to assess the likely distribution of offspring in the neighbourhood. Although our models assume that males have full information, preliminary models in which extra-pair males have more accurate information than social males predict that extra-pair mating may evolve more easily and to higher levels.

The models have for simplicity omitted several important behaviours. For example, males may attempt to pre-empt paternity losses through mate guarding or compete to sire extra-pair offspring [98]. It is in each male’s interest to protect paternity in his own nest, regardless of the cooperative benefit that follows from paternity being spread across different nests (consider the very steep drop in fitness of the social male if his female increases extra-pair mating in Fig. 2C). Whether mate guarding, frequent within-pair copulation, increased advertisement, or extra-pair mating effort evolve depends on how males best can allocate their reproductive investment [98]. Such strategies may bring the realized extra-pair paternity level in the population below that predicted by our models.

### Model predictions

Our models only caricature the complex behavioural interactions at bird breeding grounds, but even from this simplified evolutionary game several general patterns emerge. It is worth stressing that the mechanisms we propose do not preclude the simultaneous operation of ‘good genes’ effects, infertility insurance, or other mechanisms that may cause extra-pair mating or cooperation.

#### Prediction: Extra-pair mating increases fitness of whole nests and neighbourhoods

A key prediction from ‘good genes’ hypothesis is that extra-pair offspring (EPO) should have higher fitness than their within-pair (WPO) half-siblings. Conclusions from detailed population studies are variable: sometimes there is no difference between WPO and EPO or even a survival cost to EPO [99], sometimes there is a benefit to some EPO [63], [100], and in other cases EPO show more consistent benefits [28]. Meta-analyses that integrate across studies conclude that genetic benefits to EPO are weak or absent in most cases and are unlikely to be the main driver behind the widespread occurrence of extra-pair mating [33], [34].

In contrast, it follows from our mechanism that extra-pair mating improves fecundity or survival of whole nests or neighbourhoods. The most striking observation of this is not from birds but from a rodent, the Gunnison’s prairie dog (*Cynomys gunnisoni*), for which there was a direct relationship between litter size and the number of males the female had copulated with [101]. In this species, groups of multiple males and multiple females share a network of underground burrows, and females forage aboveground, frequently beyond territory borders. This could be consistent with our model for resource territories, although the paper does not report territory location for the extra-pair males. To our knowledge, bird data has only rarely been analysed for whole-nest effects in a similar way. Predator mobbing is common in tree swallows (*Tachycineta bicolor*), and here older and experienced females had more extra-pair sires [102], higher hatching success [103], and larger clutch size [104]. In dark-eyed juncos (*Junco hyemalis*) extra-pair offspring had higher fitness: sons through extra-pair offspring and daughters through increased fecundity [100], which is as expected from our theory if female extra-pair mating and male cooperative investment are heritable traits. It would be interesting to see further analyses of whole-nest and neighbourhood effects and to contrast populations of the same species differing in the level of extra-pair paternity.

#### Prediction: Neighbours dominate as extra-pair sires

We predict that extra-pair copulations should be predominantly with neighbours who can share resources, be vigilant, or help with nest defence. Where spatial patterns in extra-pair sires have been reported, neighbours dominate [27], [61], [64], [105], [106]–[109]. In song sparrows territory neighbours sired 95% of the extra-pair young in a spread-out mainland population [108] and 88% of all EPO in a dense island population [107]. There was a tenfold difference in territory size between these sites, suggesting that being a neighbour may be more important than distance itself, a conclusion also reached for reed buntings (*Emberiza schoeniclus*) [109].

The prevalence of local extra-pair sires is not readily explained by genetic benefits, although it has often been hypothesized that frequent encounters make assessment of genetic quality easier or allow more opportunity for copulation [80]. Counterarguments could be i) that extra-pair copulations with distant sires likely would reduce the probability of being detected; and ii) that several studies report higher heterozygosity for EPO with long-distance sires but no such effect for EPO with neighbours [63], [110]. The latter observation suggests that while compatibility benefits may explain extra-pair mating with non-neighbours, there is a need to look beyond genetic benefits to explain the dominance of local extra-pair sires.

#### Prediction: Cuckolding males are often cuckolded themselves

It is expected from our models that recruiting contributions from neighbouring males may enhance fitness of a female’s brood even if she is socially paired with a high-quality male. This aligns with the common observation that males who are successful at gaining extra-pair paternity are no better than the rest at defending paternity in their home nest [64], [106], [108], [111], which is not easily explained by ‘good genes’ hypotheses.

#### Prediction: Males show ‘dear enemy’ effects during the breeding season

Many territorial species are less aggressive towards neighbours than strangers [112], and such a ‘dear enemy’ effect is a direct outcome of our resource territory model. The model predicts a ‘dear enemy’ effect in the period from fertilization to fledging, while the rest of the time there might be intense competition over mates, fertilizations, and resources. This was found in the skylark (*Alauda arvensis*), where males showed no ‘dear enemy’ effect during settlement and pair formation early in the breeding season, reduced levels of aggression against neighbours but not strangers in the middle of the breeding season, while aggression towards neighbours increased again later when fledglings became independent [113]. This indicates that the ‘dear enemy’ effect is not linked to familiarity with neighbours *per se*, but that the presence of offspring, potentially extra-pair, might cause it (20% of offspring in the skylark population were extra-pair [113]).

There are two additional twists following from our hypothesis. First, the dear enemy effect is predicted mainly in the model where territories combine breeding and resources, which was found also in a literature review [112]. Second, our theory predicts that males should reduce aggression against neighbours more than females would, and in the same review the ‘dear enemy’ effect was shown most often in males, sometimes in both sexes, and only rarely in females only [112].

#### Prediction: Extra-pair paternity is correlated with predation risk

Females may not be equally successful in recruiting a cooperative network under all conditions. Low predation risk might reduce extra-pair mating and male-male cooperation through two routes. Firstly, the expected benefit from male-male protection might be low when there are few predators (model for collective vigilance and defence), which would reduce the incentive for females to engage in extra-pair mating in the first place. Secondly, the expected longevity of males will likely be higher when predation is low, and males may then reduce current reproductive investments and prioritize future breeding attempts if extra-pair paternity levels are high. The prediction of reduced extra-pair paternity at low mortality rates has been documented in literature reviews [62], [114] but is also a prediction from theory that does not consider cooperative benefits [88].

Individuals might show flexible behaviours or mating strategies in response to perceived predation risk. When exposed to stuffed predators, willingness to engage in mobbing and other cooperative behaviours was increased in pied flycatchers (*Ficedula hypoleuca*) [115], [116], but it remains to be seen whether this may also correlate with extra-pair mating behaviour.

#### Prediction: Extra-pair paternity is correlated with breeding density

It follows from our hypothesis that a positive within-species relationship between breeding density and extra-pair mating can be expected, as has been observed for several species [109], [117], [118]. Figure 4B shows increasing fitness with increasing group size, indicating that if females can choose where to settle they might prefer dense neighbourhoods. In species where males settle first, this may lead males to choose territories within aggregations because they attain higher mating success. A positive correlation between breeding density and extra-pair mating has been documented within some bird species [1], [119], [120], but not between species [92], [119]. Such correlations may arise through several mechanisms, for example, extra-pair mating has been viewed as a cost of sociality and not a cause for it, exemplified by the ‘opportunity hypothesis’ where extra-pair mating is more common in dense breeding aggregations because females interact with more potential mates [80]. Related is the ‘hidden lek’ hypothesis [121], suggesting a female preference for breeding in aggregations because it allows them to better compare male quality. From both these hypotheses one would expect strong mating skew in breeding aggregations, but studies on birds often suggest a more equal partitioning of extra-pair offspring, in line with our hypothesis. No reproductive skew was found in aggregations of least flycatchers (*Empidonax minimus*) [122], but direct effects in the form of more alarm calling [123] or lower predation rates in central nests was found in some studies [124] although not all [125]. In yellowthroat warblers (*Geothlypis trichas*) there was lower variance in male reproductive success in dense breeding areas [61]. Interestingly, there was increased production of both within-pair and extra-pair young in dense neighbourhoods [61]. In bank swallows large colonies had positive effects on offspring survival because predator mobbing was more efficient [82]. It would be interesting to see further comparisons of cooperative behaviours and extra-pair mating between high- and low-density breeding sites.

#### Prediction: Sex-specific division of labour

As a consequence of extra-pair mating a male’s fitness incentives are spread across the neighbourhood whereas a female still has all her offspring in the nest. The distinction between maternity certainty and paternity uncertainty suggests a new evolutionary basis for sex-specific division of labour, where we predict that males are more likely to cooperate towards public goods than females. It has been noted that mobbing often is performed predominantly by males [60], [84], but none of the adaptive reasons for predator mobbing listed by Curio [54] provide adequate explanations for why there should be sex-specificity. A particularly interesting example of male-male cooperation is the calling network among red-winged blackbird males [53]. Using seven different call types, males continuously echo the background call they hear from other males to signal that all is clear [52], but change signal if there is danger or disturbance. The new signal is picked up and repeated over the entire neighbourhood, and due to this vigilance network females can forage more efficiently [126]. Song matching, whereby males incorporate elements of neighbours’ songs in their own, is also known from other songbirds [127] and may serve a similar function. From this perspective it makes evolutionary sense that alarm calls uttered by females more often are directed at her offspring to make them hide or be silent, whereas alarm calls by males more often are broadcast wider [128].

#### Prediction: Cooperative behaviours are extra common during breeding

Lima [48] reviewed anti-predator behaviours in breeding birds and noted that cooperative behaviours are abundant but that most are diffuse, have received little attention, and are poorly understood. This is puzzling, given that sociality and cooperative behaviours are particularly difficult to explain during breeding as birds often are territorial and compete for mates and resources.

Studies on red-winged blackbirds [129] and great tits (*Parus major*) [130], [131] found positive effects of breeding with familiar neighbours, i.e. between individuals who had been neighbours also during a previous breeding season. These studies further suggested that the positive effect may be related to cooperative behaviours in the neighbourhood. In both species, familiar neighbours were more likely to join in predator mobbing [129], [130]. In great tits, the strongest effects on reproduction occurred when a female had many familiar male neighbours, that is, between the individuals who ‘seal the deal’ over extra-pair mating and cooperative investment also in our model [131]. In addition, there was a small positive effect on nest success (avoidance of nest predation) if also the neighbourhood males were familiar with each other.

### Systemic effects and future directions

This paper presents a simple mechanism with far-reaching consequences: where maternity certainty incentivizes each female to focus care towards offspring in her social nest, paternity uncertainty and a potential for offspring in several nests incentivize males to invest in communal benefits and public goods. In this section we evaluate some of the systemic effects of our hypothesis and consequences beyond birds and beyond social monogamy.

#### Extra-pair mating in the light of kin selection

Kin selection theory [8], [20], [132] often considers relatedness between breeders or between breeders and helpers to explain evolutionarily stable levels of cooperation. In this context, extra-pair mating reduces relatedness in family groups and may therefore erode the basis for cooperation [6], [133]. Our models do not challenge the result that cooperative breeding in kin groups becomes more unstable with extra-group mating. Where we disagree is that this would have effects for cooperation in general, an inference that seems to follow e.g. from Cornwallis et al. [6] using ‘cooperative breeding’, for which their conclusion is valid, interchangeably with ‘cooperation’, which is a much broader phenomenon.

In our models patterns of relatedness emerge from mating strategies [134] so that the distribution of kin differs between male and female breeders. Although this alters incentives and pay-offs towards favouring male-male cooperation, the invasion analyses emphasize how male-male interactions are flavoured both by conflict over fertilizing mom’s babies as well as cooperation to benefit dad’s ‘maybes’ wherever in the neighbourhood they might be.

#### Synergy with other mechanisms for evolution of cooperation

Cooperation theory has extended the parameter region in which cooperation can be stable by including biologically plausible mechanisms such as individual recognition [11] or lack thereof [135], interaction networks [13], individual variation [136], assortative interactions [14], choice of cooperative partner [16], [137], social standing [15], self-regard [138], and reward or punishment [139]. Our models differ from classic cooperation models by allowing the cooperative investment to be a continuous trait [135], [137], by letting pay-offs be gradual and emerge from ecological interactions, and by including several types of players that differ in their characteristics and motivation. This adds ecological realism to cooperation models [22], but was also essential for understanding that cooperative dynamics may underlie an ecologically well-studied problem such as extra-pair mating.

Although reciprocity, kin selection, and group-level selection are not included in our models, these mechanisms may act in synergy with extra-pair mating and make it easier for cooperation to evolve to the high levels observed in breeding populations. In principle, a small effect of ‘good genes’ or infertility insurance could favour an initially low rate of female extra-pair mating, which could trigger and entrain selection towards cooperative behaviours and higher extra-pair mating. If males can recognize extra-pair offspring or divert attention to nests that more likely contain them, the behavioural interactions become less diffuse and more reciprocal [44], [115]. If males signal cooperativeness and females base their choice of extra-pair mates on such a signal, then competitive altruism may add momentum to the evolution of cooperation [16], [137]. Once established, female extra-pair mating and male-male cooperation could spread through group-level selection [10]. It is interesting to note that among birds, relative brain size, which could be relevant for individual recognition of cooperative partners or for manoeuvring the complex behavioural games when extra-pair mating is involved, is correlated with social monogamy but not with genetic monogamy [140].

#### Individual differences: A methodological challenge but evolutionarily potent

Our models do not include individual differences among males or among females, but in the wild individuals differ from each other in age, experience, condition, plumage, parasite load, etc. Obviously, these differences may lead to variation in reproductive success and context- or state-dependent mating strategies.

With no individual variation, the models predict an equal share of paternity among males and an equal share of cooperative benefits to all females and their offspring. With variation in individual characteristics these patterns will likely change, but to predict the direction is not straightforward. Assume that males vary in their resource holding power, then males capable of defending rich territories may obtain a higher share of extra-pair matings than the average male in his neighbourhood, and individual differences may be a source of variation in male reproductive success. In contrast, differences may equalize among females, as females with poor mates or territories may gain access to additional resources and protection through extra-pair mating. There is also increasing awareness of how variation in individual quality may mask trade-offs or relationships between individual performance across traits [141], as some individuals excel in pretty much everything [142] or because motivation differs [143], [144].

With individual differences, a basis for mate preferences arises. If females actively seek extra-pair mates who invest more heavily in cooperation, this may enhance or stabilize cooperative investments in public goods [16], [137]. Females not only benefit from cooperative extra-pair males, but also from care-giving social mates, which may cause females to prefer different traits in extra-pair compared to social mates [145]. Finally, there may be conflict between choosing a care-giving social partner and choosing a cooperative neighbourhood for breeding, as cooperative males may settle assortatively to boost the effects of their cooperative investments.

#### Relevance beyond birds and social monogamy

To extend the relevance to other taxa there is a need to consider taxon-specific differences in reproductive physiology and the mode of fertilization. In fish, fertilization is most often external, which allows the male to better assess paternity as he can know whether he released gametes in proximity of the spawning female and often whether other males were around too. With higher paternity certainty males can identify conditions when it is safe to invest in care for a single brood, and exclusively paternal care is widespread among fish [146]. One interesting manipulation of paternity spread in fish is found in the cichlid *Julidochromis transcriptus*. Here females spawn in wedges between rocks and so use the topography of the environment to distribute paternity: large males who provide safety only can fertilize the outer eggs whereas the innermost eggs can only be reached by a much smaller male, who later cares for the brood [147].

With internal fertilization there is greater paternity uncertainty but it also allows a greater share of the parental investment to be physiologically linked to the female. Internal fertilization in fish is often related to live-bearing and having big offspring with little direct care from males. Female birds produce large eggs while males may share incubation and subsequent feeding and protection until fledging. In mammals the period of exclusively maternal care is further prolonged by lactation, and male provisioning of dependent young is much rarer. Interestingly, the prevalence of multi-male mating for viviparous species is roughly the same when comparing fishes to mammals to reptiles [68] and to birds [1].

In birds, the combination of paternity uncertainty and only limited reproductive investment being inextricably linked to female physiology pave the way for social monogamy where males and females may share several of the tasks involved in reproduction. In contrast, investments are often split temporally in fish where females produce eggs while males may provide care, or by task in mammals where males may help with vigilance or defence but not so often with direct offspring provisioning or care [56], [148].

The considerations above imply that the core mechanism of paternity uncertainty and male-male cooperation may actually be reversed in fish. In the many species with external fertilization and where males build nests and brood eggs, our theory suggests that when females lay eggs in several nests they gain diffuse maternity incentives across the neighbourhood.

In primates and humans males hunt and share large game and engage in vigilance [83], warfare, and defence [56], [149]; activities that all resemble public goods. In some human societies there are even cultural practices by which extra-pair mating is ritualized following communal work days [150] or collective hunting [151], suggesting that extra-pair mating may have influenced evolution of cooperation and sociality in our own species.

The right end-point of Figures 1A and 4A are analogous to group breeding as paternities in each nest are divided evenly among the breeding males. Among primates there are many group-living species with frequent multi-male mating and paternity spread. In these, within-group mating may enable peaceful and productive group dynamics by favouring male cooperative behaviours [56], [89]. The logic of our mechanism may even be extended to extra-group mating to obtain between-group friendliness [56], [152], [153].

The privileged status of genetic explanations for female choice, suggested by Darwin [154] and canonized by Trivers [24], may have led to a historical downplay of ecological benefits as a possible explanation for extra-pair mating. Our suggested mechanism reinstates ecology and direct benefits in this perspective, and links variation in predation risk or breeding habitat to differences in male and female competitive and cooperative behaviours. By redistributing paternity and fitness incentives across a neighbourhood, female-driven extra-pair mating may have been a central step towards evolution of the high levels of cooperation observed in many species. In addition to being a mechanism for the evolution of cooperation, our model suggests that extra-pair mating may also be driving the evolution of breeding aggregations, and together cooperation and aggregation are cornerstones of sociality. The theory presented here may thus provide one missing step towards explaining the highly developed sociality, what is often termed pro-sociality, in many primates and humans [89].

## Supporting Information

Supporting Information S1 **Supplementary methods.** Contains detailed model description, full table of variables and parameters, and supporting figures.
(PDF)

Supporting Information S2 **Comparison with Prisoner’s Dilemma.** Compares the dynamics of the resource territoriality model with a standard Prisoner’s Dilemma game.
(PDF)
