# Supplementary material for: Extra-Pair Mating and Evolution of Cooperative Neighbourhoods
Source: PLoS One. 2014 Jul 2;9(7):e99878. doi: 10.1371/journal.pone.0099878 (PMC4079691; doi:10.1371/journal.pone.0099878)
Supplement: Supporting Information S1 — Supplementary methods. Contains detailed model description, full table of variables and parameters, and supporting figures. (PDF) [file pone.0099878.s001.pdf]

Supplementary Methods

Extra-pair mating and evolution of cooperative neighbourhoods

Sigrunn Eliassen and Christian Jørgensen

Original article appeared in PLoS ONE 2014.
Correspondence to: sigrunn.eliassen@bio.uib.no

Contents

Model for resource territories S1
Table 2. Overview of notation and standard parameter values (both models) S2
Fig. 7. Assumptions of resource defence model S3
Fig. 8. Dynamics of extra-pair paternity at different longevities (resource territory model). S7
Fig. 9. Sensitivity of extra-pair paternity to parameter variation (resource territory model). S8
Model for collective vigilance and anti-predator defence S9
Fig. 10. Dynamics of extra-pair paternity at varying group sizes (resource territory model). S10

Model for resource territories

This model describes a breeding system where males and females form socially monogamous pairs and where both pair members may provide care for offspring at the nest. Central assumptions are:

i) both males and females provide care only at their social nest;
ii) males allocate their effort between territorial defence, which monopolizes resources, and care at the nest;
iii) females divert a fixed effort to egg production and the remaining reproductive investment to care;
iv) offspring survival depends on parental care and resources monopolized by the social male.

Parental care

We denote the care contribution from females and males c_f and c_m, respectively, and assume that the relative effect of care investments on offspring survival depends on each parent's contribution:

$$f = c_\mathrm{f}^{\gamma} + c_\mathrm{m}^{\gamma} \ , \qquad (1)$$

where $0 < \gamma \leq 1$ gives care effects that increases linearly or with diminishing returns as care investments rise. Results do not differ qualitatively if a linear relationship is assumed compared to a diminishing returns function.

Resource defence and territoriality

Males fight for access to breeding territories that contain resources important for growth and survival of offspring. Investment in territorial advertisement and defence, d, determines the amount of resources that males are able to monopolize, which has the effect q, on offspring survival. Resource competition is modelled as a tug-of-war, where the share of resources that one male monopolizes depends on his territorial investment relative to that of his neighbour. If parents monopolize abundant food resources, offspring survival will often depend more on other factors, such as protection from predators, which corresponds to declining marginal benefits of monopolizing even further resources as the territory grows in size. We therefore assume that the effect of resource availability on offspring survival scales as a power function with exponent $0 < \alpha \leq 1$. Although our standard parameter represents diminishing returns ($\alpha = 0.7$) the results do not differ qualitatively if a linear relationship is assumed.

Consider a focal male who invests d' in territorial behaviour while his neighbour invests d (throughout, prime will denote a focal individual that uses a slightly different strategy compared to the rest of the population). The benefit of resources for the focal male is then:

$$q(d' \mid d) = \left(\frac{d'}{d' + d + l} \right)^{\alpha} \ , \qquad (2a)$$

while his neighbour benefits as

$$q(d \mid d') = \left(\frac{d}{d' + d + l} \right)^{\alpha} \ . \qquad (2b)$$

Table 2. **Overview of notation and standard parameter values.** *Resource territory model; **Collective vigilance and defence model.

Symbol	*Description*	*Value*
**Strategy variables**		
c_f	Maternal care (subscript ‘f’ indicates female)	
c_m	Paternal care (subscript ‘m’ indicates male)	
d	Male investment in territorial defence	
k	Male investment in collective vigilance and defence	
x	Rate of extra-pair paternity	
**Functions**		
f	Effect on offspring survival of parental care	
g	Total benefit for offspring survival of being in a group	
m_f, m_m	Total annual mortality rate	
OSR	Sex ratio when pairs are formed at start of breeding season	
p_f, p_m	Pairing probability of males and females	
q	Effect on offspring survival of resources defended in territory	
r_f, r_m	Total reproductive investment	
s_f, s_m	Survival of parents from pair formation until end of care	
y_f, y_m	Expected number of breeding attempts	
w_f, w_m	Expected fitness, proportional to lifetime production of fledglings	
**Ecological and life-history parameters**		
a	Cost of aggregating	0.04
b	Proportion of annual adult mortality during breeding season	0.3*; 0.0**
h	Rate at which group benefits increase with cooperative investment	0.5
l	Competitive pressure from floaters per resource area	0.1
m_0	Annual basal mortality rate	Varied
m_r	Annual mortality rate due to reproductive investment at $r = 1$.	0.1*; 0.25**
n	Number of breeding pairs in neighbourhood	Varied
r_0	Female basal reproductive investment in eggs	0.6*; 0.3**
u	Summed group investment in cooperation at which the effect of collective defence increases most rapidly	2.0
v	Proportion of m_0 experienced from fledgling until first breeding	0.5
α	Exponent in function q (effect of resources)	0.7
β	Exponent of mortality increase for reproductive investment	3*; 1**
γ	Exponent in function f (effect of care)	0.7

Here l is the background pressure from floaters or others who compete for access to resources in the territory. A positive value of l requires a minimum investment from males to defend a territory.

Reproductive investment

We denote the total reproductive investment of females and males by r_f and r_m, respectively. For females, r_f includes any initial egg investment r_0 and maternal care at the nest c_f:

$$r_f = r_0 + c_f \quad . \tag{3a}$$

For males, we assume territorial behaviour d consumes time and energy and males risk injury from fights or predation; territorial behaviour is thus in conflict with providing care c_m at the nest (Hegner *et al.* 1987; Qvarnström 1997). For simplicity, we assume that these are exclusive activities and that there is a linear trade-off between them:

$$r_m = d + c_m \; . \tag{3b}$$

The effect of care and male territorial investment is shown in Fig. 7*A*,*B*, and their combined effect on fitness is illustrated in Fig. 7*C*.

Combined effect of resources and care on fitness

Resources that are secured through territory defence influence the efficiency of care investments from parents, and it is the combined effect that determines offspring production. We assume that all females have the same clutch size and that each female lays all her eggs in her social nest (no brood parasitism). Consider

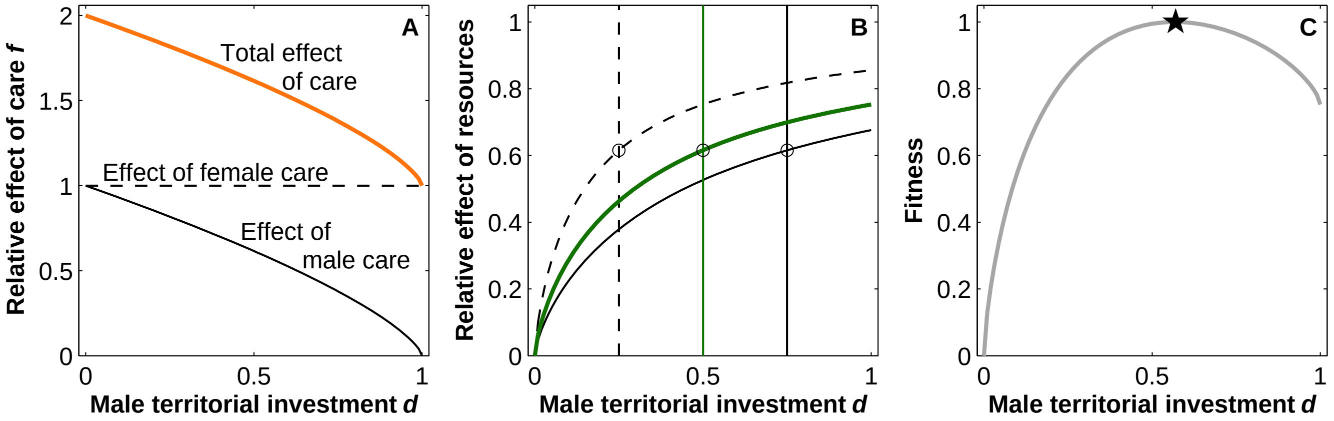

Fig. 7. Assumptions of resource territory model. (*A*) As male territorial investment *d* increases, males have less time for care as $c_m = r_m - d$, and the relative benefit of male care drops (solid black line). As female care c_f is assumed constant (dashed black line), the total effect of care, $f = c_f^\gamma + c_m^\gamma$, will drop when males invest more in territorial defence *d*. (*B*) With increasing territorial investment *d*, a male will monopolize more resources, which benefit offspring in his nest. The thick green line shows the effect of variation in a focal male's defence strategy *d* given that the opponent invests 0.5 (vertical green line). The solid (dashed) black line assumes that the opponent invests more (less) in territorial defence. The open circles indicate that a male can monopolize the same share of resources as his opponent as long as they both have the same investment level (in this example the pressure from floaters is $l = 0.0$). (*C*) Fitness is the combined effect of care (panel A) and resources (panel B; assuming that opponent invests $d = 0.5$) and peaks (★) at an intermediate territorial investment. (Parameters: $\alpha = \gamma = 0.7$; $n = 2$; $r_0 = 0$; $r_f = r_m = 1$.)

a female who only lives for a single breeding season; female fitness w_f is then the expected offspring production in her nest. We model this as the product of the relative effect of care and the relative effect of resources (Fig. 7*C*):

$$w = f(c'_m, c_f)q(d' \mid d) \quad (4)$$

The internal dynamics of the model becomes clearer if we focus on one male with a slightly different investment strategy (denoted by prime) $r'_m = c'_m + d'$. Offspring survival in his social nest is

$$\begin{aligned} w^{WP} &= f(c_f, c_m)q(d' \mid d) \\ &= \left(c_f^\gamma + c_m^\gamma\right)\left(\frac{d'}{d' + d + l}\right)^\alpha \end{aligned} \quad (5)$$

We see that increasing territorial defence d' will increase the share of resources with a positive effect on offspring survival in the nest, but it will simultaneously decrease investment in paternal care c'_m, which has a negative effect.

We are interested in finding the evolutionarily stable strategy (ESS), in this case the male strategy for care and defence that cannot be invaded by another strategy that does better. We do this by finding the selection gradient on the investment strategy of the focal male given that his neighbour's reproductive investment is $r_m = d + c_m$, then update the neighbour's strategy in the direction of the positive selection gradient, and repeat the procedure until the gradient vanishes.

Extra-pair paternity and sex-specific fitness

With no extra-pair mating in the population, a female and her social mate have the same fitness $w_f = w_m = w^{WP}$. This changes, however, if the male loses paternities in his own nest or sire offspring at a neighbour's nest. Assume first that all males gain and lose paternity with the same probability *x*, so that the number of offspring that a male sires is on average the same, it is only the distribution between nests that changes. For a focal male, fitness now depends on fitness in his social nest w_f^{WP} and in the neighbour's nest w_f^{EP}, and how within-pair and extra-pair offspring are distributed:

$$w_m = (1 - x)w_f^{WP} + x w_f^{EP} \quad . \quad (6)$$

Note that a change in the male's care strategy will only affect fitness in his own nest, w_f^{WP}, while a change in territorial behaviour will redistribute resources and have consequences for his offspring in both nests (within-pair and extra-pair). In the following we will study how optimal male reproductive strategies are affected by extra-pair paternities, and use specific models that are tailored to study complementary perspectives of the general description above.

Two pairs (Fig. 1 in main text)

First, we assume that males and females have a fixed total investment in reproduction so that $r_f = c_f = 1$ and $r_m = c_m + d = 1$. The effect of these strategies on fitness is given in Eq. 5.

Consider a focal male who invests differently from his neighbour. If he invests more in territorial defence ($d > d'$), then he will control a larger share of the total resource, as $q(d' \mid d) > q(d \mid d')$. His within-pair offspring will benefit from more resources, but he simultaneously provides less care, so a higher d' will evolve only if the positive effect from extra resources is higher than the cost of reduced care.

If there are extra-pair paternities ($x > 0$), then the focal male will also affect the fitness of his extra-pair offspring in the neighbouring nest. While a higher d' secures more resources for his within-pair offspring, his extra-pair offspring in the neighbouring nest will suffer from lower resource availability. As before the male invests less in care due to his higher territorial activity which affects his within-pair offspring only. As a consequence, the incentives for males to monopolize resources drop as x increases. With increasing x, reduced territorial aggression (lower d') frees time that leads to increasing care c_m.

Analytical solution to the model in Fig. 1

For the simplified cases where $r'_m = r_m = 1$ and $l = 0$, a focal male with a slightly different strategy has fitness [from Eqs. (5) and (6)] equal to:

$$\begin{aligned} w_m(d' \mid d) &= (1-x)w_f^{WP} + x w_f^{EP} \\ &= (1-x)\left(1+(1-d')^{\gamma}\right)\left(\frac{d'}{d'+d}\right)^{\alpha} \\ &\quad + x\left(1+(1-d)^{\gamma}\right)\left(\frac{d}{d'+d}\right)^{\alpha} \end{aligned} \tag{7}$$

The notation for male fitness reflects that the focal male uses defence strategy d' and care strategy $c'_m = 1-d'$. The partial derivative with respect to d' gives the change in the focal male's fitness in a population of males investing c_m and d. An evolutionary stable strategy exists when $\partial w_m / \partial d' = 0$ and $d' = d$. Noting that we are not interested in the solution $d' = 1$ when males provide no care and fitness is at a local minimum, and further simplifying by setting $\alpha = \gamma$, we get (Eq. 8):

$$(4x-3)(1-d)^{\gamma} + d\,(1-x)(1-d)^{\gamma-1} + 2x - 1 = 0$$

which can be solved by Newton's method. Because the analytical solution only works for simple cases, we used numerical simulations to find the ESS.

Numerical implementation

For the numerical solutions we use invasion analysis and the logic of adaptive dynamics theory (Metz *et al.* 1992; Dieckmann & Law 1996). This approach assumes that a monomorphic population follows what is termed a resident strategy. We then consider a single or rare mutant with a slightly different value for the strategy (denoted by prime), and the selection gradient is found as the ratio of the fitness of the mutant strategy relative to the fitness of the resident strategy. If the selection gradient is positive, the resident strategy is replaced with a new strategy that has shifted the value a small step in that direction. This is repeated until the best response is the strategy itself, which gives the ESS.

In small groups such as the two pairs considered here, the assumption made by adaptive dynamics that there are no population feedbacks does not hold. We therefore perform an invasion analysis based on a similar type of comparison: we first simulate a resident two-pair constellation where both males follow the strategy d to find the resident fitness $w_m(d)$ of the focal male, then we perturb the focal male's strategy by a small value $d' = d + \Delta d$, and find the fitness $w_m(d')$ of this mutant assuming that all the other players use the resident strategy:

	**Resident simulation**		**Invasion simulation**	
	**Focal pair**	**Neighbours**	**Focal pair**	**Neighbours**
**Female strategy**	x	x	x	x
**Male strategy**	d	d	d'	d
**Male fitness**	$w_m(d)$	–	$w_m(d')$	–

The selection gradient is then $[w_m(d')-w_m(d)]/w_m(d)$. We repeated this procedure iteratively until the selection gradient vanished, implying that the ESS is found. The resulting male strategy d^* can be viewed as the best response given the female EPP strategy x.

Above we assumed a fixed female x, but we can study the evolutionary dynamics of the EPP rate assuming it is a female-driven strategy. For each x, we first simulate the resident population by finding the best male response d^*. Here both pairs are identical, so male and female fitness are the same. Then we

perturb the focal female's strategy to $x' = x + \Delta x$, but now we need to find the best responses of both the within and extra-pair male:

	Resident simulation		Invasion simulation	
	**Focal pair**	**Neigh-bours**	**Focal pair**	**Neigh-bours**
**Female strategy**	x	x	$x' = x + \Delta x$	x
**Male strategy**	d^*	d^*	d^*_{WP}	d^*_{EP}
**Female fitness**	$w_{\mathrm{f}}(x)$	–	$w_{\mathrm{f}}^{\mathrm{WP}}(x'; d^*_{\mathrm{WP}} \mid d^*_{\mathrm{EP}})$	–

The selection gradient on x is then

$$[w_{\mathrm{f}}^{\mathrm{WP}}(x'; d^*_{\mathrm{WP}} \mid d^*_{\mathrm{EP}}) - w_{\mathrm{f}}(x)] / w_{\mathrm{f}}(x) \ . \tag{9}$$

The gradients can be visualized as invasibility plots (Fig. 2A), and to create these we varied the x of the focal female within the whole range of possible values of x to illustrate a wider evolutionary landscape.

The gradients in Fig. 2 are based on the assumption that both the within-pair and extra-pair male respond facultatively (in ecological time) to changes in a female's extra-pair mating behaviour. Since the benefit to females is linked to direct ecological effects of male cooperative behaviour, there will be no selection gradient on female propensity to have EPP if males are indifferent to alterations in x. Each male's knowledge about within- and extra-pair paternity needs not be accurate, however, nor do males have to compensate fully. WP males and EP male may also be unequally informed about changes in the focal female's mating behaviour. Empirical studies indicate that males assess paternity based on female behaviour, rather than direct assessment of relatedness (reviewed in Kempenaers & Sheldon 1996). When male responses are based on imperfect information, the selection gradient on female EPC behaviour is weaker, but has the same evolutionary endpoint as in our model. If we assume that the EP male, who actually mates with the neighbouring female, is better informed than the cuckolded social mate, the selection gradient is slightly stronger. It is only in the situation where the WP male has accurate information and the EP male is poorly informed that the EPP endpoint is affected – the evolutionary stable strategy is then slightly lower EPP rates than originally predicted.

Note that higher EPP rates can also result from other mechanisms, including selection on male extra-pair mating strategies, female choice targeting cooperative males, or differences in offspring production between groups (see also *Discussion* in main text).

Effects of longevity on reproductive investment, shown in Fig. 5

During a lifetime, reproductive success depends on offspring production in each mating season and the number of years an individual breeds. Above, male and female reproductive investments were only allocated to different activities within the same breeding season. Cuckolded males are, however, predicted to reduce their current effort if they can expect higher paternity in future reproductive bouts (Mauck et al. 1999; Houston & McNamara 2002). This is based on the common observation that reproductive investment in one season influences future reproductive events, for instance by reducing the chance that a parent will survive to the next breeding season (Williams 1966). We also assume there are ecological factors independent of reproductive investment that result in a baseline mortality of m_0. In addition, mortality increases with the total reproductive investment r (here without subscript because the same relationship is used for both females and males), which gives an annual mortality rate:

$$m = m_0 + m_{\mathrm{r}} r^{\beta}, \tag{10}$$

where m_{r} scales the relative cost of reproduction in terms of higher mortality and β is the exponent of this trade-off.

In this model, male reproductive strategies are flexible, and both c_{m} and d can evolve to take any value. For females we assume a fixed baseline investment in eggs, r_0, but care investments c_{f} and hence total reproductive investments r_{f} can evolve to any value. For both males and females, total reproductive investment is traded against survival (as in Eq. 10), both in terms of reduced survival during the parental care period (Eq. 11) and survival costs between breeding seasons (Eq. 14). Survival through the breeding season depends on the fraction b of the annual reproduction-related mortality that adults experience prior to chick fledging:

$$s(r) = \exp\left(-b(m_0 + m_{\mathrm{r}} r^{\beta})\right) \ . \tag{11}$$

Including the probability that a male or a female may die during the parental care period, the total benefit of parental care is:

$$f(c_f, c_m, d) = s(r_f)c_f^{\gamma} + s(r_m)c_m^{\gamma} \;, \qquad (12)$$

remembering that $r_f = r_0 + c_f$ and $r_m = d + c_m$. The amount of resources defended by a territorial male and its effect on offspring survival now depend on whether the focal male (and his neighbours) survives through the breeding season:

$$q(d', c'_m \mid d, c_m) = \left(\frac{d' s(r'_m)}{d' s(r'_m) + (d + l) s(r_m)} \right)^{\alpha} \;. \qquad (13)$$

We assume that fledglings experience a mortality rate that is proportional to the baseline mortality, so that survival probability to the first breeding season is $\exp(-vm_0)$. The expected number of breeding seasons for an individual that invests r each reproductive season is then (Eq. 14):

$$y(r) = \exp(-vm_0) \sum_{i=1}^{\infty} \exp\left(-(m_0 + m_r r^{\beta})(i-1)\right) = \frac{\exp(-vm_0)}{1 - \exp\left(-(m_0 + m_r r^{\beta}\right)}$$

Here i refers to age group. For simplicity, we assume that an individual follow the same investment strategy in all breeding seasons.

Both life-time expectancy and resulting population sex ratios are emergent traits that result from male and female reproductive investment strategies. When females invest more in reproduction than males, they have lower survival probability, which result in a male biased sex ratio. The sex ratio at the onset of the breeding season (*OSR*) determines the pairing probability:

$$OSR = \frac{y(r_f)}{y(r_m)} \;. \qquad (15)$$

Note that the *OSR* only depends on the sex ratio in the resident population because a mutant is assumed to be so rare that it does not affect population-level traits. Assuming social monogamy, male and female paring probability, p_m and p_f respectively, are given by:

$$p_m = \begin{cases} OSR & \text{for} \quad y(r_m) \ge y(r_f) \\ 1 & \text{for} \quad y(r_m) < y(r_f) \end{cases} \;, \qquad (16a)$$

$$p_f = \begin{cases} 1 & \text{for} \quad y(r_m) \ge y(r_f) \\ 1/OSR & \text{for} \quad y(r_m) < y(r_f) \end{cases} \;. \qquad (16b)$$

For a mutant female with reproductive strategy $r'_f = r_0 + c'_f$, fitness is:

$$w_f(c'_f) = y(r'_f) p_f f(c'_f, c_m, d) q(d, c_m \mid d, c_m) \;. \qquad (17)$$

Similarly, given x, fitness for a male with reproductive strategy $r'_m = c'_m + d'$ is (Eq. 18):

$$w_m(c'_m, d') = y(r'_m) p_m \cdot \left[(1-x) f(c_f, c'_m, d') q(d', c'_m \mid d, c_m) \right. \cdot \left. + x f(c_f, c_m, d) q(d, c_m \mid d', c'_m) \right]$$

For each EPP value x, we consider the success of a mutant female c'_f and males strategies c'_m and d' relative to that of the resident strategies in the population. For each iteration, we make a small change to the resident strategies according to the selection gradients. For each x, c_f^*, c_m^*, and d^* is found where a mutant is no longer able to invade a population following the resident strategy (adaptive dynamics).

When the basal reproductive investment of females r_0 is high, females depend more on male care and they generally evolve a higher reproductive investment than males, which results in male-biased sex ratios. In our baseline scenario, an intermediate basal investment $r_0 = 0.6$ results in similar optimal male and female reproductive investments at EPP $x = 0$. As EPP increases, r_m^* decreases relative to r_f^*, causing a male-biased sex ratio in most scenarios.

For each basal mortality rate m_0, we find the ESS strategies for males and females along a gradient of EPP rates. Because males are expected to care at the nest where their paternity is highest (Parker & Schwagmeyer 2005), we constrain EPP rates at $x \le (n-1)/n$.

For each m_0 the evolutionary outcome for male and female investment strategies c_f^*, c_m^*, and d^* determines the expected number of reproductive seasons in each case. We consider the average female fitness along the EPP gradient starting from $x = 0$. There will be a positive selection gradient for higher x whenever the average fitness of a mutant female with strategy $x' = x + \Delta x$ is higher than female fitness in pairs following the resident strategy. For these comparisons we consider fitness of paired females only, as this is the subset of the population that expresses the extra-pair mating trait. When there is a positive selection gradient to a maximum fitness value, we consider c_f^*, c_m^*, d^*, and x^* to be the convergent stable strategies. Extra-pair paternity, mating strategies, and fitness consequences are shown for different longevities in Fig. 8. Sensitivity of the EPP prediction to parameter variation is shown in Fig. 9.

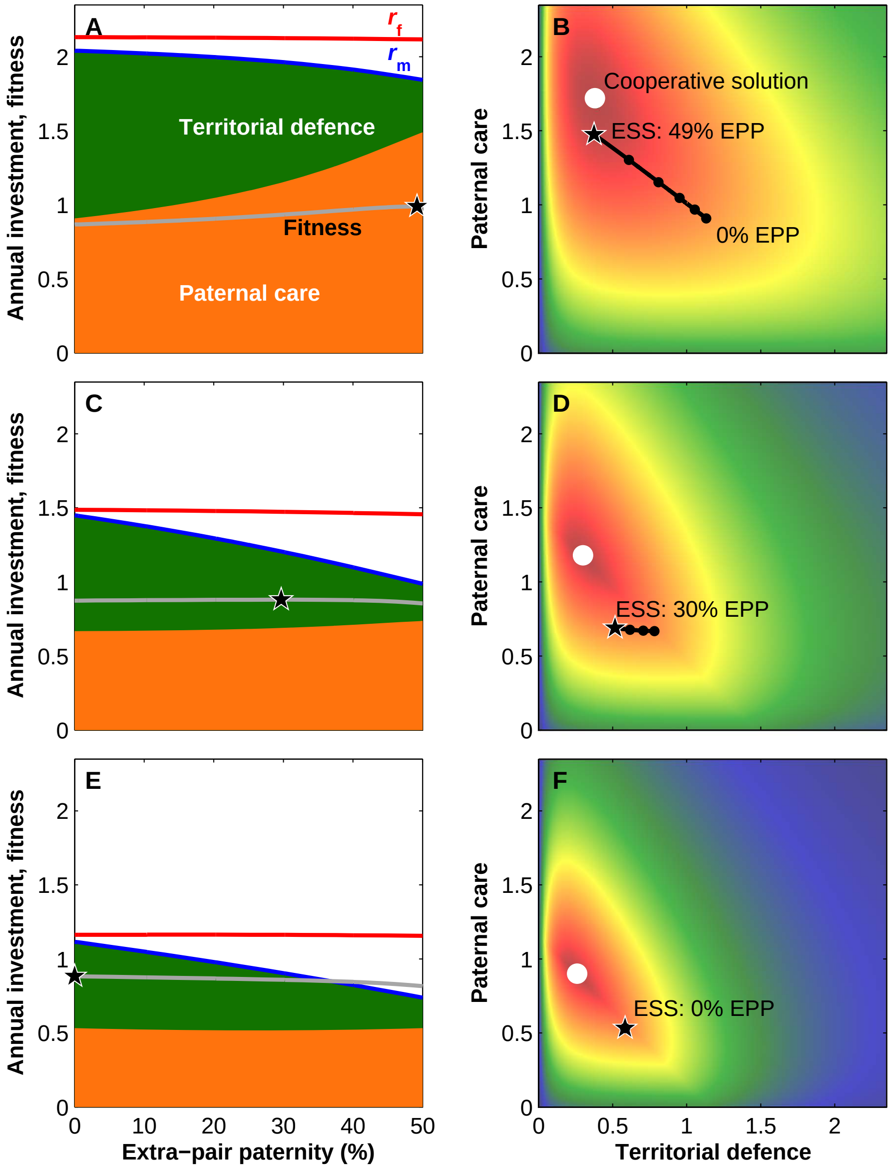

Fig. 8. Dynamics of extra-pair paternity at different longevities (resource territory model). (*A*,*C*,*E*) Best response strategies for male reproductive investment r_m (blue line) and how it is partitioned between paternal care c_m (orange area) and territorial defence d (green area) for different levels of EPP (x-axis). Total female reproductive investment r_f (red line) is also shown. The peak (★) of the resulting fitness curve (grey line; normalized to 1 for the cooperative solution) yields the evolutionarily stable strategy and the EPP level that will evolve in the population. (*B*,*D*,*F*) Fitness landscapes for male reproductive investment strategies, red colours indicate higher fitness. The cooperative solution (white circle) is the strategy that gives the highest average fitness. The solid black line is the best male response when individual-level selection is acting, and indicates the male strategy that maximizes individual fitness given a level of EPP (circles indicate each 10% value; star is the evolutionarily stable strategy). Longevity is 0.5 years (panels *A* and *B*; $m_0 = 1.6$), 1 year (panels *C* and *D*; $m_0 = 0.79$), and 2 years (panels *E* and *F*; $m_0 = 0.38$). Other parameters as in Fig. 1, except $v = 0.5$.

A social neighbourhood of more than two pairs

We now extend the model to include interactions among several breeding pairs in a local neighbourhood. A neighbourhood can be aggregations of territories within a limited area or a continuous network of adjacent territories. The benefit to offspring of resources monopolized by a focal male $q(d'|d;n)$ when there are n males competing for all the resource in a neighbourhood is

$$q(d' \mid d; n) = \left(\frac{d'n/2}{d' + (n-1)d + nl/2} \right)^{\alpha} . \qquad (19)$$

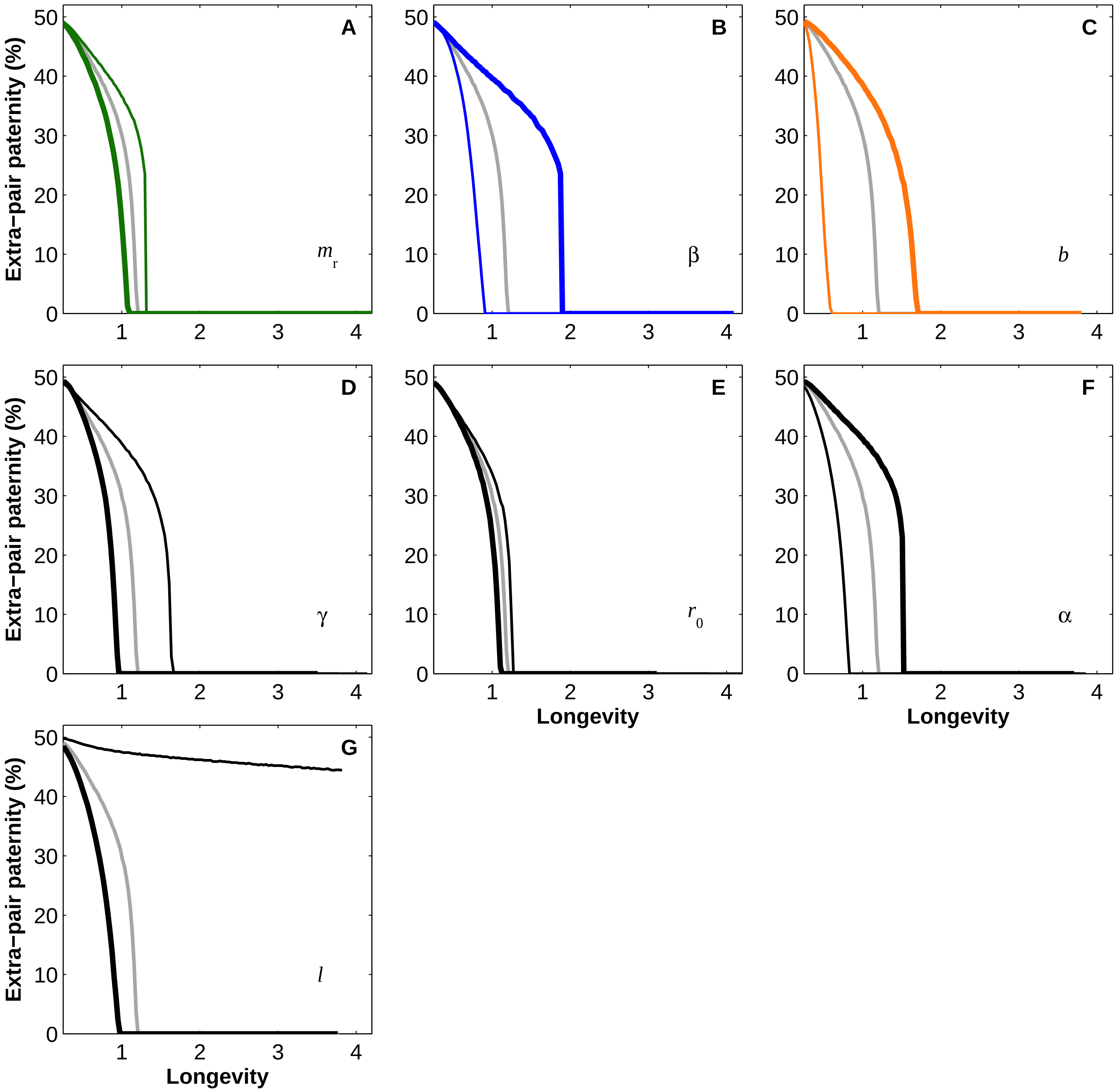

Fig. 9. Sensitivity of extra-pair paternity to parameter variation (resource territory model). The grey line in all panels is the standard parameter set as in Fig. 1. (*A*) Mortality cost of reproductive investment m_r (thin line $m_r = 0.05$, thick line $m_r = 0.2$). (*B*) Scaling exponent β for mortality cost of reproductive investment (thin line $\beta = 2$, thick line $\beta = 4$). (*C*) Proportion of annual adult mortality incurred during the breeding season (thin line $b = 0.1$, thick line $b = 0.5$. (*D*) Scaling of benefit of care (thin line $\gamma = 0.4$, thick line $\gamma = 1.0$). (*E*) Female basal reproductive investment (thin line $r_0 = 0.3$, thick line $r_0 = 0.9$). (*F*) Scaling of benefit of resources (thin line $\alpha = 0.4$, thick line $\alpha = 1.0$). (*G*) Pressure from floaters l (thin line $l = 0.02$, thick line $l = 0.4$). (v =0.5.)

Note that for comparison, we let the total resource scale with the number of competing males, with a per capita area of 1/2 as for the two pair model ($n = 2$). Here we consider the lifetime reproductive success of a focal male who invests d' when all the neighbouring males invest d. The competitive pressure from floaters scales with the size of the resource area. As before, offspring production in the focal nest depends on care benefits and resource availability, which gives the within-pair female fitness:

$$w^{\mathrm{WP}} = f(c'_{\mathrm{m}}, c_{\mathrm{f}})q(d' \mid d; n) \ . \tag{20}$$

For an average EPP level of x, we assume that a male has an equal probability of siring extra-pair offspring in any of the neighbouring nests. Male reproductive output is given by

$$\begin{aligned} w_{\mathrm{m}}(c'_{\mathrm{m}}, d') &= (1-x)w_{\mathrm{f}}^{\mathrm{WP}} + (n-1)\frac{x}{n-1}w_{\mathrm{f}}^{\mathrm{EP}} \\ &= (1-x)w_{\mathrm{f}}^{\mathrm{WP}} + xw_{\mathrm{f}}^{\mathrm{EP}} \\ &= (1-x)f(c'_{\mathrm{m}}, c_{\mathrm{f}})q(d' \mid d; n) \\ &\quad + xf(c_{\mathrm{m}}, c_{\mathrm{f}})q(d \mid d'; n) \end{aligned} \quad (21)$$

When we consider multiple breeding seasons, monopolized resources now also depend on survival:

$$q(d', c'_{\mathrm{m}} \mid d, c_{\mathrm{m}}; n) = \left(\frac{d's(r'_{\mathrm{m}})(n/2)}{d's(r'_{\mathrm{m}}) + \left((n-1)d + nl/2\right)s(r_{\mathrm{m}})} \right)^{\alpha} \quad (22)$$

which then replaces the relationship in Eq. 13. EPP predictions and mating strategies for different group sizes are shown in Fig. 10.

Model for collective vigilance and anti-predator defence

We now turn the focus to the need for protection from predators. In a dense and connected neighbourhood there are potentially more individuals that can detect an approaching predator or join a mob to defend the nest (Caro 2005). We therefore introduce a strategy k for investment in cooperative anti-predator behaviours, interpreted as the frequency by which a male will join a mob or give an alarm call, or the time he spends on vigilance. We assume that the benefit to offspring of being in a neighbourhood depends on the average male investment $\bar{k}$ in cooperative anti-predator behaviours and the neighbourhood size n, following a sigmoid function

$$\frac{1}{1+\exp(-h(n\bar{k}-u)} \quad (23)$$

where h determines the slope and u the point at which the cooperative benefits are increasing at the fastest rate. Aggregations of nests may also increase conspicuousness to predators, lead to conflicts among neighbourhood members, increase competition for resources, or increase pathogen load. We consequently assume a cost of aggregating relative to breeding in a solitary nest of exp[–a(n–1)] where a is a parameter for the aggregating costs. Combining the costs and benefits of living in a neighbourhood, the effect g on offspring survival will for most parameters peak at an intermediate neighbourhood size (Fig. 3; main text):

$$g(\bar{k}, n) = \frac{\exp\left(-a(n-1)\right)}{1+\exp\left(-h\left(n\bar{k}-u\right)\right)} \,. \quad (24)$$

In a resident population where all individuals use the same strategy, the average is $\bar{k} = k$. For the sake of notation, let $\bar{k}' = [k' + (n-1)k]/n$ denote the group's average investment when a focal male uses a slightly different strategy k'. We also redefine the total male reproductive investment so that it is now $r_{\mathrm{m}} = c_{\mathrm{m}} + k$.

The benefit of care is modelled as in the model for resource defence (Eq. 1), so the combined effect on female fitness of cooperative anti-predator defence and care is now:

$$w_{\mathrm{f}} = f(c_{\mathrm{f}}, c_{\mathrm{m}})g(\bar{k}, n) \,. \quad (25)$$

Female fitness w_{f} equals male fitness if there is no extra-pair mating in the population.

Introducing extra-pair paternity

Assume now that all males loose a fraction x of the paternities in their own nest and have an equal probability of siring offspring in other nests within the group (we assume that extra-pair matings are restricted to the social neighbourhood). For a mutant male in a focal pair, reproductive output depends on fitness in his social nest ($w_{\mathrm{f}}^{\mathrm{WP}}$) and in the other nests in the group ($w_{\mathrm{f}}^{\mathrm{EP}}$) (Eq. 26):

$$\begin{aligned} w_{\mathrm{m}}(c'_{\mathrm{m}}, k') &= (1-x)w_{\mathrm{f}}^{\mathrm{WP}} + xw_{\mathrm{f}}^{\mathrm{EP}} \\ &= (1-x)f(c_{\mathrm{f}}, c'_{\mathrm{m}})g(\bar{k}', n) + xf(c_{\mathrm{f}}, c_{\mathrm{m}})g(\bar{k}', n) \\ &= g(\bar{k}', n)\left[(1-x)c'^{\gamma}_{\mathrm{m}} + xc^{\gamma}_{\mathrm{m}} + c^{\gamma}_{\mathrm{f}}\right] \end{aligned}$$

Begin again with the assumption that the total reproductive investment of males r_{m} is fixed. A focal male who invests more in cooperative defence than the other males in the group ($k' > k$) will consequently invest less in care, which affects all the young in his nest. The benefit of improved collective defence, on the other hand, will benefit all offspring in the group equally. As a consequence, a male that has a large proportion of his offspring outside the nest (relatively more extra-pair young) will experience a lower cost to benefit ratio when he start increasing his cooperative investment. Consequently, males have a higher motivation for cheating, by investing less in cooperative group defence when they have all their offspring in a single nest than when paternity is spread around the neighbourhood.

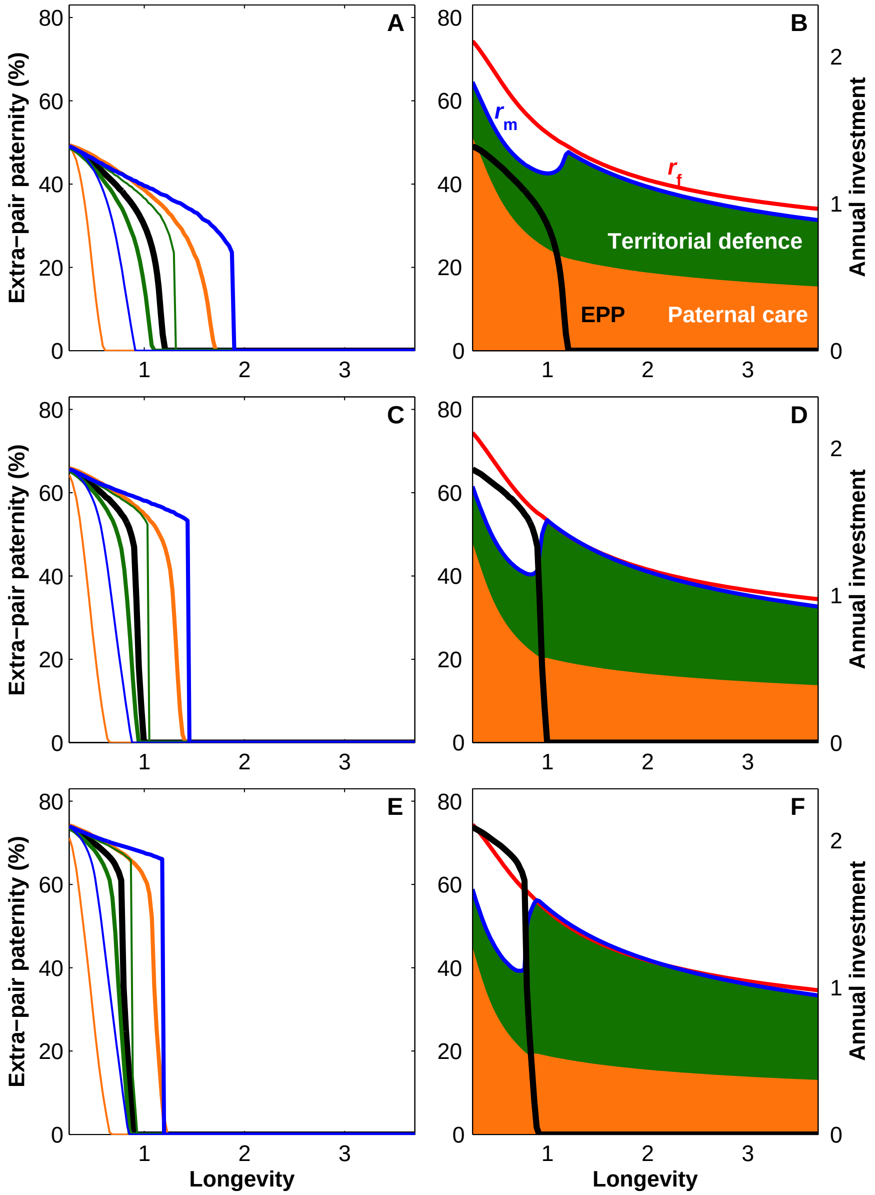

Fig. 10. Dynamics of extra-pair paternity at varying group sizes (resource territory model). (*A*,*C*,*E*) Predicted EPP levels given increasing longevity. Thick black line is standard parameters (as in Fig. 3*A*). Coloured lines show sensitivity to key ecological parameters: mortality cost of reproductive investment (green; thin line $m_r = 0.05$, thick line $m_r = 0.2$); scaling exponent for mortality cost of reproductive investment (blue; thin line $\beta = 2$, thick line $\beta = 4$); proportion of mortality incurred prior to hatching (orange; thin line, $b = 0.1$; thick line $b = 0.5$). (*B*,*D*,*F*) Corresponding changes in reproductive strategies for the standard scenario (EPP given by thick black line). Male reproductive investment r_m (blue line) and how it is partitioned between territorial defence d (green area) and paternal care c_m (orange area). Female reproductive investment r_f is also shown (red line). Group size is $n = 2$ pairs (panels *A* and *B*), $n = 3$ pairs (panels *C* and *D*), and $n = 4$ pairs (panels *E* and *F*); model otherwise as in Fig. 1 (except $v = 0.5$).

Introducing longevity

When males and females reproductive investment can be allocated between seasons their survival probability between breeding seasons is assumed to depend on current reproductive investment as described in Eqs. 11 and 14. Lifetime reproductive output in a monomorphic population where all individuals follow a resident strategy is (Eq. 27):

$$w_f = w_m =$$
$$\begin{cases} y(r_f)\, f(c_f, c_m, k)\, g(n, \bar{k}) & \text{for} \quad y(r_m) \geq y(r_f) \\ y(r_m)\, f(c_f, c_m, k)\, g(n, \bar{k}) & \text{for} \quad y(r_m) < y(r_f) \end{cases}$$

For a focal male having proportion x extra-pair offspring, fitness is:

$$w_\mathrm{m} = \frac{y(r'_\mathrm{m})}{y(r_\mathrm{m})}\left((1-x)w_\mathrm{f}^\mathrm{WP} + x w_\mathrm{f}^\mathrm{EP}\right) \ . \qquad (28)$$

As with the model for resource defence, we use invasion analysis to find the ESS for the two male strategies c_m and k at different EPP rates x and different group sizes n. We compare the fitness of mutant strategies to the fitness of resident strategies when in a group where all males invest c_m and k assuming a fixed reproductive investment of females. The evolutionary outcomes are found by adaptive dynamics.

At a given baseline mortality rate m_0 we consider the average fitness of the best male and female strategies at different group sizes moving along the EPP gradient starting from $x = 0$. If there is a positive gradient to a maximum fitness value, we consider the corresponding c_m^*, k^* and x^* to be the convergent stable strategies for an optimal group size n^*.

References

Caro T (2005) *Antipredator defenses in birds and mammals* (University of Chicago Press, Chicago, IL) p 592.

Dieckmann U, Law R (1996) The dynamical theory of coevolution: a derivation from stochastic ecological processes. *Journal of Mathematical Biology* 34:579-612.

Hegner RE, Wingfield JC (1987) Effects of experimental manipulation of testosterone levels on parental investment and breeding success in male house sparrows. *The Auk* 104:462-469.

Houston AI, McNamara JM (2002) A self-consistent approach to paternity and parental effort. *Phil Trans R Soc B* 357:351-362.

Kempenaers B, Sheldon BC (1996) Why do male birds not discriminate between their own and extra-pair offspring? *Anim Beh* 51:1165-1173.

Mauck RA, Marschall EA, Parker PG (1999) Adult survival and imperfect assessment of parentage: effects on male parenting decisions. *Am Nat* 154:99-109.

Metz JAJ, Nisbet RM, Geritz SAH (1992) How should we define fitness for general ecological scenarios. *TREE* 7:198-202.

Parker GA, Schwagmeyer PL (2005) Male “mixed” reproductive strategies in biparental species: Trivers was probably right, but why? *Am Nat* 165:95-106.

Qvarnström A (1997) Experimentally increased badge size increases male competition and reduces male parental care in the collared flycatcher. *Proc R Soc Lond B* 264:1225-1231.

Williams GC (1966) Natural selection, the costs of reproduction, and a refinement of Lack's principle. *Am Nat* 100:687-690.
