# Supplementary material for: Extra-Pair Mating and Evolution of Cooperative Neighbourhoods
Source: PLoS One. 2014 Jul 2;9(7):e99878. doi: 10.1371/journal.pone.0099878 (PMC4079691; doi:10.1371/journal.pone.0099878)
Supplement: Supporting Information S2 — Comparison with Prisoner’s Dilemma. Compares the dynamics of the resource territoriality model with a standard Prisoner’s Dilemma game. (PDF) [file pone.0099878.s002.pdf]

Extra-pair mating and evolution of cooperative neighbourhoods

Sigrunn Eliassen and Christian Jørgensen

Original article appeared in PLoS ONE 2014.

Correspondence to: sigrunn.eliassen@bio.uib.no

Extra-pair mating compared to Prisoner's Dilemma

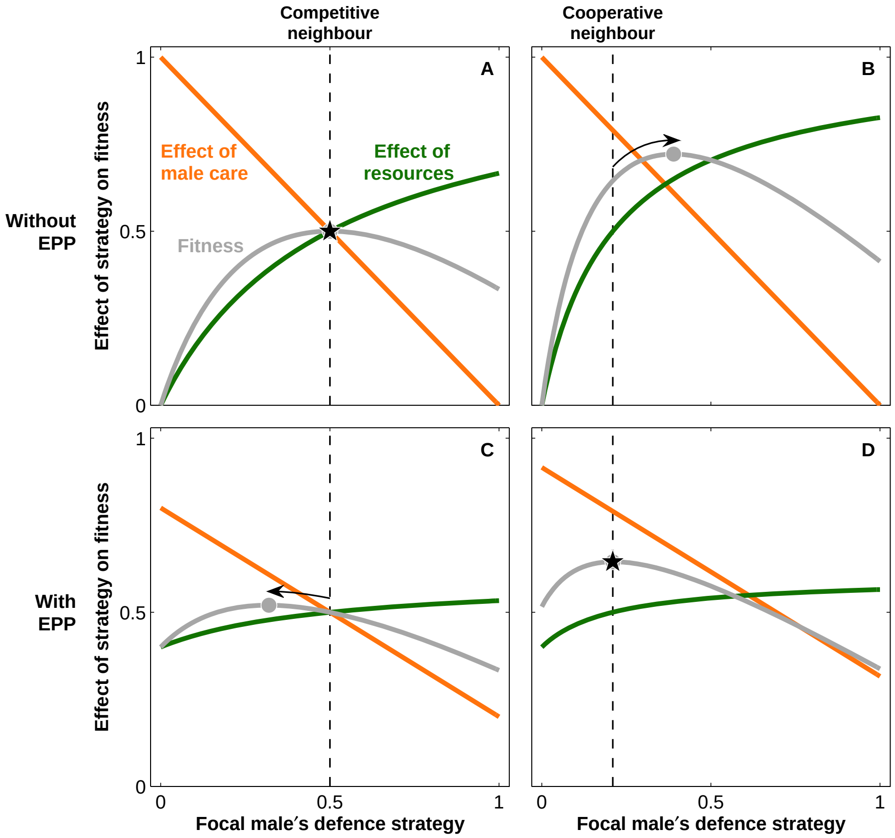

(A) In the absence of extra-pair paternity the evolutionarily stable strategy (★; ESS) where all males maximize individual fitness involves both territorial defence and paternal care at moderate levels.

(B) If the males had cooperated, they would both be better off, spending less time fighting and investing more in care. A cooperative neighbour (indicated by vertical dashed line) would, however, easily be outperformed by a cheater who gains higher fitness by being more aggressive (arrow). This can be interpreted as a one-shot Prisoner's Dilemma game, where cooperation, although better for the group as a whole, is not attained because of self-interest.

(C) Female extra-pair mating plays the important role of altering the costs and benefits of resource monopolization. Assume for simplicity that both females have the same extra-pair mating strategy so that the expected level of extra-pair paternity is the same in the two nests. Territorial defence still secures resources for a male's social nest, but now it also takes away resources from his extra-pair offspring in the neighbouring nest. In contrast, changes in care only affect within-pair offspring, but they are fewer. Thus, the curves for effects of resources and care both become less steep. Starting from the previous ESS (in panel A) selection now favours reduced investment in territorial defence and more investment in care.

(D) Because extra-pair paternity causes males to have offspring in several nests, it shifts the incentives of males from focusing on a single nest towards providing services for the neighbourhood. As extra-pair paternity increases, the ESS that maximizes individual male fitness approaches the cooperative solution: territorial defence is weaker, which frees time for paternal care in both nests and gives higher individual fitness for males and females in both pairs. [Extra-pair paternity rate is 40% in (C) and (D).]
